# Decoding the duality of GAI anthropomorphism and its joint effects—a sequential mixed-methods approach

**DOI:** 10.3389/fpsyg.2025.1615342

**Published:** 2025-11-28

**Authors:** Meng Zhang, Yang Yang, Cuiting Yu, Yimin Diao

**Affiliations:** 1 School of Business Administration, Southwestern University of Finance and Economics, Chengdu, China; 2 School of Mathematics, Southwestern University of Finance and Economics, Chengdu, China; 3 School of Economics and Management, Southwest Petroleum University, Chengdu, China

**Keywords:** generative artificial intelligence, dual anthropomorphism, failure, continuance intention, expectation confirmation mode, mixed-methods

## Abstract

The advancement of anthropomorphic generative artificial intelligence, especially in large language models and multimodal capabilities, has developed its two dimensions: functional anthropomorphism and interactional anthropomorphism. Despite this progress, prior research has predominantly emphasized interactional anthropomorphism, neglecting a holistic understanding of the dual dimensions and their combined effects. This research utilizes a sequential mixed-methods approach, starting with qualitative interviews (*n* = 15) to explore the joint effects of dual anthropomorphism. The qualitative results were incorporated into a subsequent series of experiments aimed at testing the joint effects, their underlying mechanisms, and boundary conditions. By extending the Expectation Confirmation Model (ECM), this research integrates the dual anthropomorphic features of GAI into a dynamic process that links users’ initial expectations—both cognitive and emotional—to their subsequent experiences, evaluations, and continuance intentions. This user-centered approach addresses the growing demand in IS research to focus on not only technological features but also on how these features influence user experiences. The findings provide practical recommendations to GAI service designers and deployers, offering strategies to enhance user experiences and improve the effectiveness of GAI applications.

## 1 Introduction

Anthropomorphic feature has long been considered a crucial factor influencing user attitudes and behaviors in human-computer interaction (HCI; Spatola and Chaminade, 2022; Xin and Liu, 2025). With the rapid development of technology, the anthropomorphic features of generative artificial intelligence (GAI) exhibit characteristics distinct from those traditional forms, evolving into two dimensions: functional anthropomorphism and interactional anthropomorphism (Dwivedi et al., 2023). Functional anthropomorphism refers to GAI’s ability to mimic human cognition and problem-solving to perform tasks, reflecting whether its capabilities can meet users’ cognitive needs (Alavi et al., 2024). In contrast, interactional anthropomorphism represents GAI’s ability to interact with humans in a natural, human-like manner, showing how well it satisfies users’ emotional needs (Chakraborty et al., 2024).

Despite increasing interest in AI anthropomorphism, prior research remains fragmented and unintegrated. Studies in HCI and IS have typically examined either functional or interactional aspects of anthropomorphism in isolation—focusing on performance-related competence or affective communication (Blut et al., 2021). This separation has led to three theoretical limitations. First, existing studies provide an unbalanced understanding of anthropomorphism, emphasizing emotional expressiveness while neglecting cognitive capability. Second, research has rarely examined the joint or compensatory effects of these two dimensions, overlooking how emotional engagement may buffer cognitive shortcomings or, conversely, amplify positive experiences. Third, theoretical frameworks such as the Expectation Confirmation Model (ECM) have yet to be systematically extended to account for this dual-pathway mechanism—linking cognitive and emotional expectations to user evaluations and continuance intentions in the context of GAI. Accordingly, this study seeks to bridge these gaps by developing and empirically testing an integrated framework of GAI’s dual anthropomorphism.

While GAI systems can engage in natural, human-like conversations, it remains unclear whether their outputs sufficiently address users’ informational and cognitive expectations (Alavi et al., 2024). For example, GAI may deliver engaging and witty dialog (Fui-Hoon Nah et al., 2023) yet fail to provide accurate reasoning or contextually relevant solutions (Dwivedi et al., 2023). In such cases, users’ emotional connections to GAI may influence whether they forgive or persist despite cognitive shortcomings. Accordingly, our first research question is as follows:

RQ 1: How does the dual-dimensionality of anthropomorphism jointly influence user continuance intention?

Despite the rapid progress in GAI, it continues to face major challenges (Alavi et al., 2024). Notably, its cognitive reasoning capabilities remain underdeveloped, often resulting in suboptimal performance in functional anthropomorphism (Dwivedi et al., 2023; Brendel et al., 2023). In contrast, its interactional anthropomorphism has reached a relatively mature level (Fui-Hoon Nah et al., 2023). Therefore, it is crucial to explore whether the interactional anthropomorphic features of GAI can compensate for its functional deficiencies (Dwivedi et al., 2023). Based on this, we propose the second research question:

RQ 2: How does interactional anthropomorphism work in scenarios where GAI does not exhibit sufficient functional anthropomorphism, and what are the boundaries of this effect?

To answer these questions, this research adopts a sequential mixed-methods approach that combines qualitative and quantitative methods. First, we conduct a qualitative study using semi-structured interviews to explore the joint effects of GAI’s dual anthropomorphism. A modified Expectation Confirmation Model is developed and provides a theoretical basis for formulating hypotheses. Second, we use experiments to demonstrate the proposed joint effects of the dual dimensions of GAI anthropomorphism on user continuance intention.

This study provides theoretical contributions to multiple research fields. First, it deepens the understanding of the anthropomorphic characteristics of GAI—an increasingly prominent technological feature in the current development of GAI—and its mechanisms of influence by identifying two dimensions of anthropomorphism (i.e., functional anthropomorphism and interactional anthropomorphism) and their combined effects. Second, this study responds to the current call in the Information Systems (IS) field for user-centered research by shifting the focus from the technological features embodied by GAI to the impact of these features on users’ expectations and experiences (Schuetzler et al., 2020; Kopp et al., 2022). It provides a nuanced depiction of the complex mechanisms and relationships underlying these effects. Third, this study extends the ECM by constructing a more comprehensive analytical framework. This framework integrates the two types of anthropomorphic features of technology into the dynamic process linking users’ initial expectations (including both cognitive and emotional expectations), expectation confirmation, evaluation, and continuance intention. These insights offer practical guidance for optimizing GAI design and improving user experience strategies.

## 2 Literature review

### 2.1 GAI and anthropomorphism research

With the rapid advancement of large language models (LLMs) and multimodal capabilities, generative artificial intelligence (GAI) increasingly exhibits anthropomorphic characteristics, allowing it to emulate human-like cognition, reasoning, and interaction (Li et al., 2024; Chuanyang and He, 2025). Building on a long-standing tradition in human–computer interaction (HCI) and social cognition research, anthropomorphism has been conceptualized along two complementary lines. The first, rooted in cognitive psychology and information systems theories of perceived competence (Epley et al., 2007; Blut et al., 2021), emphasizes how people attribute human-like reasoning and problem-solving abilities to intelligent systems. The second, grounded in communication and social presence theories (Nass and Moon, 2000; Waytz et al., 2014), highlights how human-like expressiveness, tone, and reciprocity elicit emotional engagement. Integrating these two perspectives, recent developments in GAI research have begun to differentiate between functional anthropomorphism—reflecting human-like cognitive competence—and interactional anthropomorphism—reflecting human-like social and emotional expressiveness (Dwivedi et al., 2023). The comparison of functional and interactional anthropomorphism is shown in Table 1.

**Table 1:** Comparison of functional and interactional anthropomorphism.

Dimension	Functional anthropomorphism	Interactional anthropomorphism
Definition	Mimics cognitive functions like reasoning, decision-making.	Mimics human-like traits like communication and emotional connection.
Underlying Mechanism	Cognitive processes, task completion.	Emotional engagement, user interaction.
Key Outcomes	Problem-solving, meeting cognitive needs.	Emotional connection, trust, satisfaction.
Example	GAI providing logical solutions to queries.	GAI responding with a friendly tone, using relatable language.

These two forms of anthropomorphism correspond to users’ dual expectations when engaging with GAI systems: cognitive needs, related to understanding, reasoning, and task accomplishment, and emotional needs, related to empathy, warmth, and connection. GAI that demonstrates a high level of functional anthropomorphism can simulate human cognition and logic, thereby satisfying users’ cognitive expectations (Kshetri et al., 2024). In contrast, GAI with high interactional anthropomorphism communicates in a natural, socially intuitive way—through verbal and nonverbal cues—fulfilling users’ emotional expectations (Chakraborty et al., 2024). The extent to which GAI meets these two types of needs shapes users’ overall experiences and evaluations (Dwivedi et al., 2023).

Although prior studies have offered valuable insights into anthropomorphism, they tend to emphasize one dimension at the expense of the other. The literature has largely focused on interactional anthropomorphism—such as appearance or conversational warmth—while giving less systematic attention to functional anthropomorphism (Blut et al., 2021; Lu et al., 2024). This imbalance has resulted in a fragmented understanding of the dual nature of anthropomorphism in intelligent systems. Furthermore, previous research has seldom examined how the two dimensions operate jointly. While interactional anthropomorphism has been shown to enhance user trust and satisfaction, its potential to compensate for functional deficiencies remains underexplored (Zhou and Zhang, 2024).

Importantly, generative AI provides a novel context in which these two dimensions are not merely additive but dynamically interdependent. Because LLM-based systems generate both reasoning and expressive outputs through the same generative mechanisms, functional and interactional anthropomorphism can co-evolve during real-time human–AI interaction. This dynamic interdependence gives rise to new phenomena—such as augmenting effects (where strong interactional anthropomorphism amplifies functional success) and compensatory effects (where interactional anthropomorphism mitigates the impact of functional failure)—that have not been theorized in traditional single-dimensional frameworks.

Moreover, technological limitations often lead to functional failures in GAI applications, such as reasoning errors or hallucinations, which may cause users’ cognitive expectations to be violated (Dwivedi et al., 2023; Brendel et al., 2023). Yet little is known about how interactional anthropomorphism might buffer users’ reactions under such conditions. Neglecting these mechanisms constrains our understanding of how anthropomorphism influences users’ attitudes and continuance intentions when expectations are not met (Yao and Xi, 2024). Therefore, examining the joint and dynamic effects of functional and interactional anthropomorphism—particularly in contexts of performance inconsistency—provides a crucial step toward a more holistic understanding of human–GAI interaction.

In summary, while previous research in robotics and chatbots has touched on cognitive and social forms of anthropomorphism separately, this study advances the literature by integrating them into a unified dual-dimensional framework that reflects the unique cognitive–emotional duality of GAI. This integrative perspective not only clarifies the conceptual foundations of dual anthropomorphism but also establishes the theoretical basis for examining how these dimensions jointly shape users’ expectation confirmation, evaluation, and continuance intention.

### 2.2 The expectation confirmation model and anthropomorphic GAI

The Expectation Confirmation Model (ECM) has been extensively used to explain continuance intentions in human-technology interaction (Brown et al., 2014; Tian et al., 2024). The model highlights the relationship between users’ expectations, experiences, and final evaluations, where expectation confirmation reflects the alignment between expectations and experiences (Oliver, 1980). A greater degree of expectation confirmation generally results in more favorable evaluations, thereby significantly influencing continuance intentions (Bhattacherjee, 2001). Previous research has applied the ECM to a range of human-technology interaction contexts, including anthropomorphic chatbot designs and GAI settings (Chen et al., 2024). For example, Lu et al. (2024) integrated cognitive and emotional expectations to investigate the impact of anthropomorphic chatbot designs, while Nan et al. (2025) examined its applicability within GAI settings. These studies underscore the ECM’s utility in analyzing the dynamics of human-technology relationships.

The application of ECM to explore the effects of GAI’s dual anthropomorphism is both appropriate and significant. First, due to the continuous nature of HGAII, the ECM—recognized for its focus on continuance intention—is particularly well-suited for investigating the evolving, partner-like relationship between humans and GAI. Second, advancements in GAI have led to dual anthropomorphism, promoting both functional and interactional dimensions that correspond to users’ cognitive and emotional expectations (Dwivedi et al., 2023). The ECM helps clarify the roles of technological features and human expectations in HGAII (Kim et al., 2009). Third, the iterative process between humans and GAI fosters a partner-like dynamic, prompting users to project interpersonal expectations, experiences, and evaluations onto their interactions with GAI (Venkatesh and Goyal, 2010). The ECM captures the interplay between expectations, experiences, and evaluations, offering a nuanced, process-based understanding of this relationship and its technological impacts (Anderson and Sullivan, 1993). Overall, the ECM provides valuable insights into the relationship between humans and GAI.

## 3 The mixed-methods approach design

To ensure methodological rigor and provide comprehensive insights into the joint effects of GAI’s dual anthropomorphism, this study employs a sequential mixed-methods approach (Tashakkori and Teddlie, 1998). We selected this methodological approach for three reasons. The research is set within a novel technological context, focusing on the duality of GAI anthropomorphism—a concept that is difficult to conceptualize and analyze using existing research (Venkatesh et al., 2013). Second, the mixed-methods design accommodates both explanatory and confirmatory research questions, ensuring a comprehensive understanding of the joint effects of dual anthropomorphism (Venkatesh et al., 2016). Third, the IS academic community increasingly advocates for mixed-methods approaches to deepen understandings and practices of GAI agents (Dwivedi et al., 2023).

Given the developmental rationale for employing the mixed-methods design, we first conducted a qualitative study to formulate hypotheses and then tested them using a quantitative study (Hua et al., 2020; Mattke et al., 2021). In the qualitative phase, we conducted semi-structured interviews to explore the relationships among users’ diverse expectations, experiences, and evaluations of GAI’s dual anthropomorphism, extending the ECM and generating a series of hypotheses. The subsequent quantitative phase tested these hypotheses through a series of controlled experiments, ensuring external validity and robustness. By integrating qualitative and quantitative findings, this research delivers a comprehensive understanding of the joint effects of GAI’s dual anthropomorphism, along with its underlying mechanisms and boundary conditions.

## 4 Stage 1: the qualitative study

### 4.1 Context of the study

This qualitative study investigated the joint impacts of GAI’s dual anthropomorphism, examined its underlying mechanisms, and identified the boundary conditions influencing these impacts. To achieve this, semi-structured interviews were conducted to gather detailed, context-rich data on user expectations, interactive experiences, and continuance intentions within the HGAII context (Ullman et al., 2024). The study had four main objectives: (1) to investigate the range of user expectations in HGAII; (2) to explore the joint effects of dual anthropomorphism on users’ continuance intentions; (3) to investigate how interactional anthropomorphism influences users’ continuance intentions when functional anthropomorphism fails; and (4) to identify key factors that shape the influence of interactional anthropomorphism when functional anthropomorphism fails.

### 4.2 Data collection

ChatGPT, a leading GAI product known for its advanced capabilities and multimodal interactivity, served as the study’s focal technology (Küchemann et al., 2024; Gessinger et al., 2025). ChatGPT demonstrates anthropomorphic traits across both functional dimensions, such as learning and reasoning, and interactional dimensions, including voice and appearance. These features, along with its capacity to establish collaborative dynamics with users in professional and personal contexts, render it an ideal platform for studying the joint effects of GAI’s dual anthropomorphism (Yang et al., 2025).

Participants were selected based on two criteria: (1) willingness to share detailed interactive experiences to enhance data richness, and (2) diversity in demographic and professional backgrounds to improve result representativeness (Song et al., 2019). Our qualitative study included 15 respondents whose demographic information and details of their interaction with GAI are presented in Appendix A.

**Figure 1:**
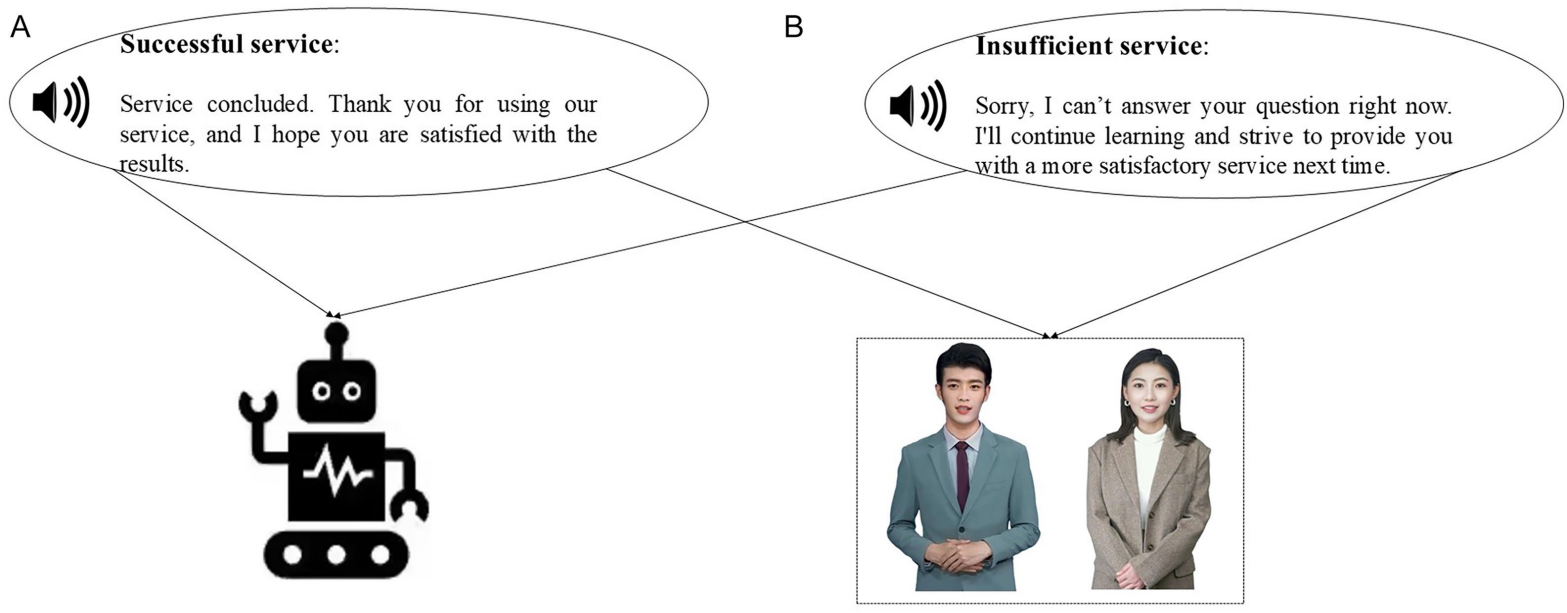
The video material used in the qualitative study. **(A)** Low-interactional-anthropomorphism. **(B)** High-interactional-anthropomorphism.

To reduce recall bias and elicit immediate, authentic responses regarding GAI’s dual anthropomorphism, the interviews incorporated experimental manipulations (Jussupow et al., 2021). Participants were exposed to video stimuli illustrating varying levels of interactional anthropomorphism after scenarios of both successful and failed functional anthropomorphism. Scenarios of high interactional anthropomorphism included digital personas with lifelike voices and appearances, whereas low-interaction anthropomorphism scenarios utilized mechanical voices and minimal visual elements (see Figure 1).

This qualitative study, conducted both in-person and online, followed a structured five-part framework: (1) an introduction setting out the background and procedures of the study; (2) questions about participants’ initial expectations of ChatGPT; (3) experience of success or failure in using ChatGPT in terms of functionality; (4) exposure to videos illustrating varying degrees of interactional anthropomorphism after each question-answer interaction (to control for gender bias, participants were randomly assigned to view either male or female digital personas); and (5) semi-structured interviews addressing participants’ responses to both the question-answer experiences and the interactional anthropomorphism videos.

The interview protocol consisted of four main sections: (1) before interacting with ChatGPT, participants were asked about their expectations of interacting with ChatGPT; (2) after experiencing successful and failed functional anthropomorphism, participants shared their continuance intentions at different levels of interactional anthropomorphism; (3) after functional failures, participants reflected on how high-interactional anthropomorphism influenced their continuance intentions; and (4) participants identified key boundary factors that shaped the effects of interactional anthropomorphism when functional anthropomorphism failed. Interviews were conducted iteratively until no new codes or themes emerged; saturation was reached after 13 interviews, and two additional interviews were conducted to confirm the stability of the thematic structure. This process enhances the credibility and completeness of our qualitative findings. See Appendix B for more details.

### 4.3 Data analysis

Grounded in the ECM framework, our data analysis focused on four key dimensions: (1) participants’ initial expectations toward of ChatGPT; (2) their continuance intentions across different interactions involving dual anthropomorphism; (3) the mechanisms through which interactional anthropomorphism influences continuance intentions when functional anthropomorphism fails; and (4) the key factors influencing the effects of interactional anthropomorphism in scenarios of failed functional anthropomorphism. The analysis process involved three phases, combining inductive and deductive coding for a thorough examination of the data. It is noteworthy that, to ensure professionalism and objectivity, all professionals engaged in the data analysis workflow within the Information Systems (IS) domain maintained independence from this study. These experts also possess specialized expertise in qualitative research methodologies.

In the first phase, two coders independently analyzed each interview transcript, focusing on four key aspects of participants’ experiences with ChatGPT: expectations, continuance intentions, underlying mechanisms, and boundary conditions. To mitigate researcher bias, each coder maintained a reflexive research journal to record assumptions, reflections, and emotional reactions throughout the coding process. This participant-centered and reflexive approach ensured that findings were grounded in authentic user experiences, enhancing the credibility and transparency of the results.

In the second phase, a third coder synthesized and reconciled the codes generated in the first phase, organizing similar codes, addressing discrepancies, and refining the coding framework. Any disagreements were resolved through collaborative discussion, and when necessary, reviewed by an external scholar to ensure inter-coder reliability. Member checking was then conducted by sharing coded summaries with selected participants to verify the accuracy and representativeness of the interpretations. An audit trail—including coding files, memos, and decision logs—was maintained to ensure analytical transparency and replicability.

**Figure 2:**
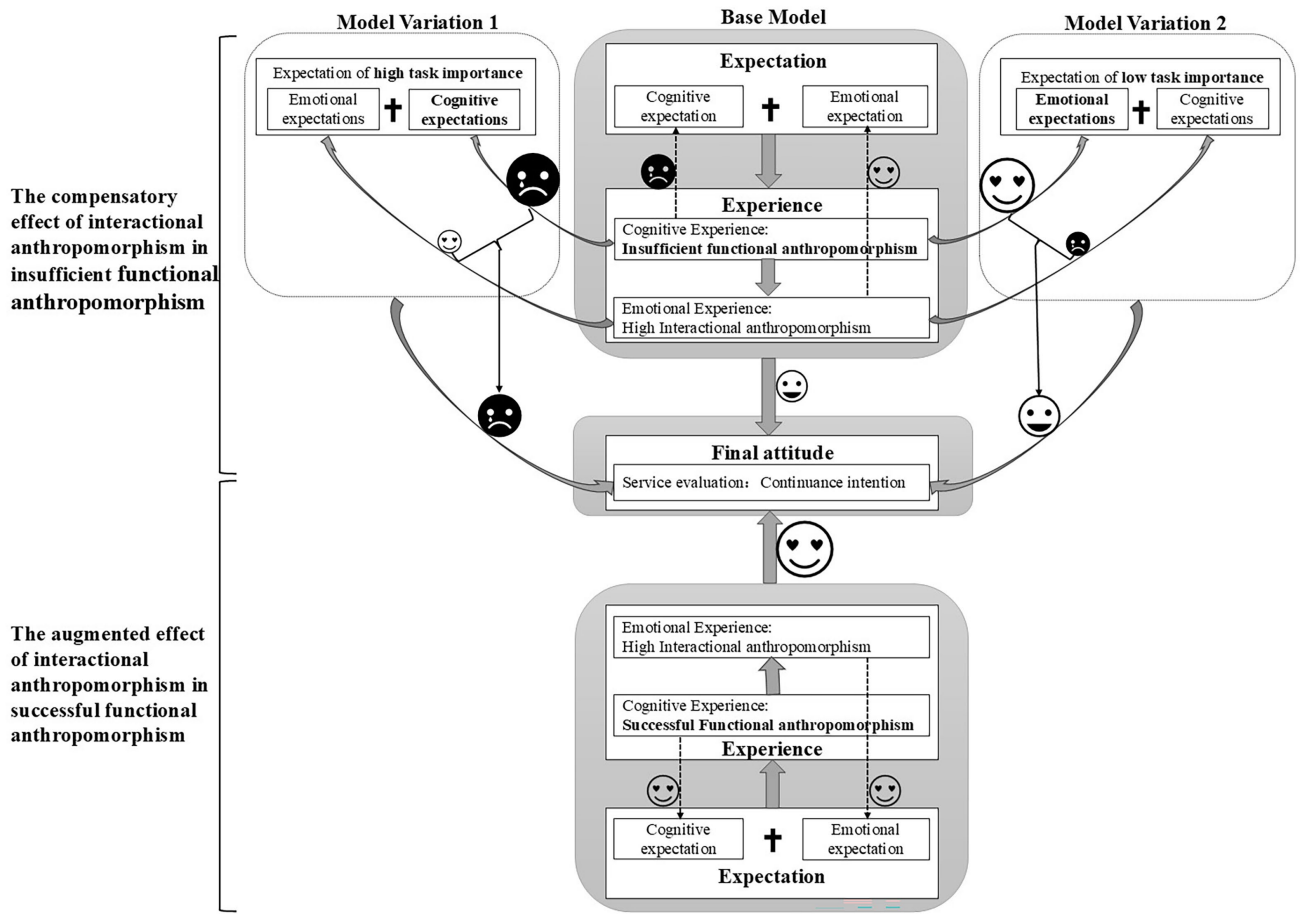
An extended ECM framework derived from the qualitative research.

In the third phase, the consolidated codes were used to validate and deepen the analysis of the four themes identified in Phase 1. Stratified purposive sampling across participants’ age, occupation, and usage levels helped minimize selection bias and ensure data diversity. During analysis, we also differentiated between stimulated responses (elicited from scenario-based videos) and natural usage experiences to avoid contextual distortion. The resulting themes provided a deeper understanding of the joint effects of GAI’s dual anthropomorphism. To further refine and validate these findings, three scholars in the field of HCI reviewed the thematic structure, leading to the development of an extended ECM framework (see Figure 2).

### 4.4 Findings

The interview study led us to the following findings: (1) when interacting with GAI, users form expectations based on both cognitive and emotional needs, which significantly influence their experience and future usage intentions; (2) the functional and interactional anthropomorphic features of GAI interact, rather than working independently, to shape user experience. Specifically, if functional anthropomorphic features fall short of expectations, interactional anthropomorphic features can compensate by reducing psychological distance and enhancing trust, helping maintain users’ willingness to continue using the system; (3) the strength of the compensatory effect of interactional anthropomorphic features varies depending on the importance of the task context.

**User expectations driven by cognitive and emotional needs**. The interview results showed that users typically have expectations regarding their cognitive and emotional needs when using GAI. Cognitive needs refer to the mental demands or expectations of users when interacting with GAI, particularly those related to processing information, making decisions, and solving problems. These requirements focus on the quality and reliability of the information provided by the system as well as how well the GAI supports users in achieving understanding or completing intellectual tasks. Emotional needs refer to the feelings or psychological aspects that users want to fulfill when interacting with GAI. These needs focus on how the interaction makes users feel, such as fostering a sense of connection, trust, comfort, and satisfaction. One interviewee said:

*“I hope that ChatGPT can give me with more diverse, specific, and clear answers, so that I can solve the problem more easily. I also hope that it can respond to our emotions and provide answers that are more attuned to my feelings to make me feel more comfortable.” (P4)*

**Interaction between functional and interactional anthropomorphic features to shape user continuance intention**. The interview study revealed that when interacting with GAI, users generally focus on both its functional anthropomorphic characteristics and its interactional anthropomorphic traits. Whether these features effectively meet their two types of needs (cognitive and emotional) significantly influences their interaction experience and subsequent willingness to continue using it. Functional anthropomorphic features of GAI indicate whether it can accurately understand users’ intentions and provide appropriate suggestions or solutions. The functional anthropomorphic features of GAI can meet users’ cognitive needs, thus having a strong impact on their experience and willingness to continue using the system. One respondent stated:

*“I asked GPT about the impact of AI on employees’ work patterns, and its response was valuable. I’m willing to continue using it… However, when I asked it to design a club activity plan, its response didn’t meet my expectations and I was disappointed.” (P1)*

Interactional anthropomorphic features refer to the human-like traits exhibited by GAI during interaction, such as tone, intonation, response speed, and likability. These features are related to whether users’ emotional needs are met and, therefore, can also influence users’ willingness to continue using the system. One respondent shared the following experience:

*“The male character in the video looks very human-like, without any robotic feel. In addition to answering questions, I think this adds extra value and would make me want to continue using this AI. The robot’s appearance, on the other hand, feels too rigid.” (P2)*

The study further points out that the functional anthropomorphic features and interactional anthropomorphic features of GAI interact dynamically, rather than functioning independently. Specifically, interactional anthropomorphic traits can either compensate for or augment the effects of functional human-like characteristics.

When the functional anthropomorphic features are insufficient (i.e., when users’ cognitive expectations are not met), the interactional anthropomorphic features play a compensatory role by reducing psychological distance and fostering relational trust. In this mechanism, the affective and relational cues conveyed by interactional anthropomorphism (e.g., empathetic tone, apologies, or social presence) help users reinterpret the unsatisfactory cognitive performance in a less negative way, thereby attenuating the impact of cognitive disconfirmation on overall satisfaction and continuance intention.

Conversely, when the functional anthropomorphic features are sufficient (i.e., users’ cognitive expectations are fulfilled or exceeded), the interactional anthropomorphic features exert an augmented effect. In this case, the cognitive and emotional confirmations work in tandem: functional anthropomorphism satisfies users’ cognitive needs (e.g., accuracy, logic, task competence), while interactional anthropomorphism fulfills affective and social expectations (e.g., warmth, empathy, responsiveness). The convergence of both confirmations leads to a stronger sense of satisfaction and a heightened willingness to continue using the system.

The combined effect of these two types of anthropomorphic features is illustrated by the following excerpts shared by the interviewees:

*“I think the fitness plan provided by GPT is great, and the female digital avatar in the video has a very gentle tone, which is pleasant to listen to. Although its response to work-related issues did not take my perspective into account, it’s not a big deal.” (P15, the augmented effect)*

*“When I asked it how to learn large models, I was not very satisfied with the answer. The robot’s response felt a bit stiff. In contrast, the male digital persona’s response showed an apology, and I felt there was at least some sincerity in it, which made me feel a bit better.” (P13, the compensatory effect)*

The compensatory effect of interactional anthropomorphism works through a sequential mechanism, namely by narrowing psychological distance which in turn enhance user trust. When functional anthropomorphism fails to make users experience cognitive confirmation, it can lead to dissatisfaction. However, high interactional anthropomorphism utilizes human-like traits to reduce psychological distance and promote a sense of closeness and connection between users and GAI. This increased closeness subsequently strengthens users’ trust in GAI, ultimately enhancing their continuance intentions. An interviewee shared her experiences interacting with ChatGPT:

*“In response to my question, GPT didn’t provide an answer that satisfied me. However, the digital persona looked quite attractive, which made me feel a sense of closeness, almost like chatting with a real person. I felt more inclined to trust what it said and was more willing to continue the conversation.” (P5)*

**Figure 3:**
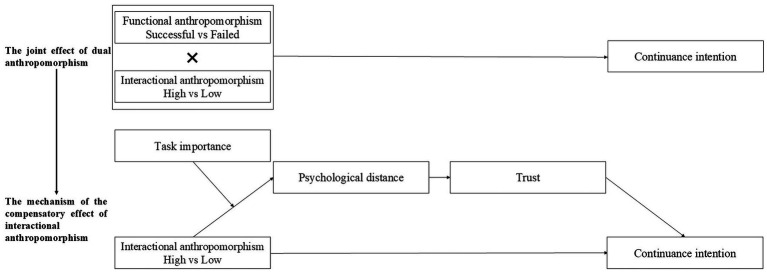
The proposed conceptual model.

**Context-dependent compensatory effect of interactional anthropomorphic features**. Research shows that task importance is a key factor influencing the strength of the compensatory effects of interactional anthropomorphism (see Appendix A). When the task that users are trying to solve is more important, they are more concerned with whether their cognitive expectations are met. In this case, the emotional fulfillment provided by interactional anthropomorphism is not sufficient to offset the negative feelings associated with unmet cognitive expectations (see the Model Variation 1 of Figure 3). When the task at hand is of low importance, users are more focused on whether their emotional expectations are met. As a result, the role of interactional anthropomorphism becomes more prominent, helping to mitigate the negative feelings associated with unmet cognitive expectations (see the Model Variation 2 of Figure 3). One respondent shared his experiences:

*“Compared to important tasks, when I ask ChatGPT less significant questions, such as recommending a movie or song, I still feel satisfied with the response, even if it doesn’t fully meet my expectations. This emotional comfort is more apparent in these less critical situations, as my expectations for the answers are lower.” (P7)*

By separating the functional and interactional anthropomorphism, as well as their respective roles in fulfilling users’ cognitive and emotional expectations, the conclusions of this qualitative study can explain how users’ actual experiences and their willingness to continue using the system are influenced by the combined impact of these two types of anthropomorphism. The underlying logic of this phenomenon can be explained based on a key derivative model of the Expectation Confirmation Model (ECM)—the Assimilation-Contrast Model (ACM; Brown et al., 2014). According to ACM, users’ evaluations are influenced by the alignment or misalignment between their expectations and actual experiences, with the degree of discrepancy modulating how they perceive the system’s characteristics and their continued use intention (Anderson, 1973). When GAI exhibits strong functional human-like characteristics that satisfy users’ cognitive expectations, interactional human-like characteristics further enhance this effect, augmenting the overall user experience and satisfaction. When functional human-like characteristics are insufficient, leading to a lower confirmation of cognitive expectations, but interactional human-like characteristics are strengthened to meet emotional expectations, the gap between the user’s overall expectations and actual experience is reduced. This, in turn, adjusts the user’s evaluation and can ultimately change their willingness to continue using the system. Additionally, in different task importance contexts, the relative importance of users’ cognitive and emotional expectations will vary (e.g., under low task importance, users place less focus on cognitive expectations). This difference alters the gap between users’ overall expectations and actual experiences, thereby affecting the strength of the compensatory effect of interactional anthropomorphism. When cognitive expectations are less emphasized, the impact of interactional human-like traits in fulfilling emotional expectations becomes more pronounced, which can lead to a stronger compensatory effect.

The findings of the above study not only validate the ECM in the context of HGAII but also extend it theoretically in the following ways. First, it integrates cognitive and emotional expectations, broadening ECM’s scope to reflect the multidimensional nature of user needs in human-AI interactions. Second, by linking functional and interactional anthropomorphism to the fulfillment of these expectations, the model highlights how high interactional anthropomorphism enhances outcomes in functional success scenarios and mitigates failures, emphasizing the interplay between technology features and user experiences. Third, it introduces a process-oriented mechanism wherein the compensatory effect of high interactional anthropomorphism operates through reducing psychological distance and enhancing trust, shedding light on how human-like traits compensate for functional shortcomings. Finally, the model identifies task importance as a key boundary condition. In low-importance tasks, users prioritize emotional expectations, allowing high interactional anthropomorphism to provide emotional confirmation that reduces the negative effects of cognitive disconfirmation. In contrast, in high-importance tasks, cognitive expectations take precedence, making functional failures more impactful and harder to mitigate. Together, these insights extend the ECM to better explain the nuanced dynamics of expectation confirmation in HGAII.

The qualitative findings here clarify key dynamics of user-GAI interaction: the roles of cognitive/emotional expectations, the compensatory/augmented effects of dual anthropomorphism, and task importance as a boundary condition. Grounded in ECM and ACM, these insights provide an empirical basis for developing a testable model and hypotheses— the focus of following section.

### 4.5 Building and hypothesizing the proposed research model

Building on the qualitative findings, this study develops a conceptual model to investigate the joint effects of GAI’s dual anthropomorphism, the underlying mechanisms, and the boundary conditions that influence these effects (see Figure 3). These effects are explained through an integration of the ECM and ACM frameworks, offering a nuanced perspective on the relationship between users and dual anthropomorphic GAI.

GAI’s dual anthropomorphism jointly influences users’ continuance intention. The joint effect primarily encompasses two aspects. First, users’ continuance intention in successful functional anthropomorphism is significantly higher than in failed scenarios. From a cognitive perspective, the degree of expectation-confirmation in successful functional anthropomorphism is higher than in failed scenarios (Dwivedi et al., 2023). Based on ECM, a higher level of expectation-confirmation is associated with a positive final evaluation (Churchill Jr and Surprenant, 1982). Therefore, users’ continuance intention in successful functional scenarios is higher than in failed ones (Dwivedi et al., 2023).

Second, high interactional anthropomorphism in GAI leads to greater continuance intentions compared to low interactional anthropomorphism by fulfilling users’ emotional expectations more effectively. Compared to low interactional anthropomorphism, GAI exhibiting high interactional anthropomorphism provides users with higher emotional expectation-confirmation, thereby leading to a higher degree of overall expectation-confirmation (Dwivedi et al., 2023; Chakraborty et al., 2024). As outlined in the ECM, greater expectation confirmation fosters more positive user evaluations (Brown et al., 2014), explaining the increased continuance intentions with high interactional anthropomorphism. Thus, users’ continuance intention is higher in the context of high interactional anthropomorphism than in low interactional anthropomorphism (Dwivedi et al., 2023).

Moreover, high interactional anthropomorphism amplifies user satisfaction in successful functional anthropomorphism (augmented effect) and mitigates dissatisfaction in failed scenarios (compensatory effect). Expectation confirmation is greater in scenarios combining successful functional and high interactional anthropomorphism than in failed functional anthropomorphism scenarios (Dwivedi et al., 2023). According to the ACM, larger discrepancies are handled differently from smaller ones (Johnston, 1995). Consequently, users’ continuance intention in successful functional anthropomorphism with high interactional anthropomorphism is stronger than in failed scenarios, exhibiting augmented and compensatory effects, respectively (Dwivedi et al., 2023).

*H*1: The interplay between functional and interactional anthropomorphism has a significant impact on users’ continuance intentions. Specifically, users continuance intention is significantly higher in scenarios of successful functional anthropomorphism compared to failed scenarios (H1a). High interactional anthropomorphism enhances users’ continuance intentions compared to low interactional anthropomorphism (H1b). In addition, high interactional anthropomorphism exerts an augmented effect, further increasing continuance intentions in successful functional anthropomorphism scenarios, and a compensatory effect, reducing the negative impact of functional failures (H1c).

The compensatory mechanism of interactional anthropomorphism operates through a sequential process involving psychological distance and trust. Psychological distance, referring to the perceived closeness or remoteness in terms of time, space, or psychology, is measured through intimacy and identity acceptance (Kim et al., 2008). Trust in GAI reflects users’ confidence in its ability to meet their needs and deliver on expectations, even under uncertain or risky circumstances (Cropanzano, 2005). Functional anthropomorphism failures lead to cognitive disconfirmation, where users’ expectations are unmet, resulting in dissatisfaction (Alavi et al., 2024). High interactional anthropomorphism, characterized by human-like appearance and communication style, fosters psychological closeness and relatability, laying the groundwork for trust (Li and Sung, 2021; Dang and Liu, 2023; Bonneviot et al., 2023). Psychological proximity increases users’ trust in GAI, which in turn motivates them to sustain their continuance intention even in the face of functional anthropomorphism failures (Lv et al., 2022).

*H*2: When functional anthropomorphism fails, high interactional anthropomorphism reduces the psychological distance between users and GAI. This reduced distance enhances users’ trust in GAI, ultimately strengthening their continuance intention.

Task importance represents the extent to which a given task is perceived as critical for achieving a user’s goals (Webster and Sundaram, 2009). Drawing on motivational and goal-criticality theories, task importance determines the relative salience of cognitive versus emotional expectations toward GAI (Locke et al., 1981; Dwivedi et al., 2023). Within the ECM framework, users form judgments by comparing their initial expectations with subsequent experiences; when the gap between the two falls within an acceptable tolerance range (Liljander and Strandvik, 1993), evaluations remain relatively stable. Task importance shapes this tolerance range and therefore moderates how interactional anthropomorphism compensates for functional deficiencies.

In low-importance tasks, users place greater emphasis on affective and relational experiences. They are more tolerant of minor functional shortcomings, allowing high interactional anthropomorphism—through warmth, empathy, and social presence—to effectively compensate for cognitive disconfirmation. Conversely, in high-importance tasks, users focus on cognitive accuracy and instrumental reliability; as the stakes increase, their tolerance for disconfirmation narrows, thereby weakening the ability of interactional anthropomorphism to offset functional failures. This reasoning aligns with recent findings that task criticality heightens goal-directed processing and reduces reliance on socio-emotional cues (Dwivedi et al., 2023; Lu et al., 2024).

*H*3: Task importance moderates the compensatory effect of interactional anthropomorphism. Specifically, when task importance is low, the compensatory effect is more pronounced; when task importance is high, the compensatory effect diminishes as cognitive expectations dominate.

## 5 Stage 2: the quantitative study

### 5.1 Overview of the experiments

The experimental scenarios were based on legal contexts, selected intentionally due to the growing public awareness of legal issues and rights protection. This choice allowed us to create scenarios that are not only relevant but also engaging for participants, thereby enhancing the ecological validity of the experimental design. We partnered with a technology retailer to gather data by engaging customers at store entrances, offering material incentives for participation.

All experimental scenarios were designed within a unified legal context, as legal consultation is a domain characterized by both high public relevance and cognitive accessibility. The growing societal awareness of legal rights makes this domain particularly suitable for testing user responses to generative AI (GAI) in consequential decision-making tasks. Focusing on a single domain helps enhance internal validity by keeping contextual factors constant, while still allowing systematic variation in task importance and realism across scenarios. Within this legal domain, we selected three specific types of disputes—civil disputes, medical liability disputes, and shopping disputes—to represent issues of different significance and emotional involvement. These are all common and socially relevant legal issues, enabling participants to easily engage with the tasks and perceive them as realistic.

We collaborated with a technology retailer to recruit participants at store entrances and provided material incentives for participation. The experiments were divided into three parts. The first part examined the joint effects of GAI’s dual anthropomorphism, assessed in Experiments 1 and 2. The second part focused on exploring the mechanisms underlying the compensatory effect of interactional anthropomorphism. The third part investigated the boundary of the compensatory effect of interactional anthropomorphism.

### 5.2 Experiments 1: the joint effect of dual anthropomorphism

#### 5.2.1 Interactional anthropomorphism stimuli

**Figure 4:**
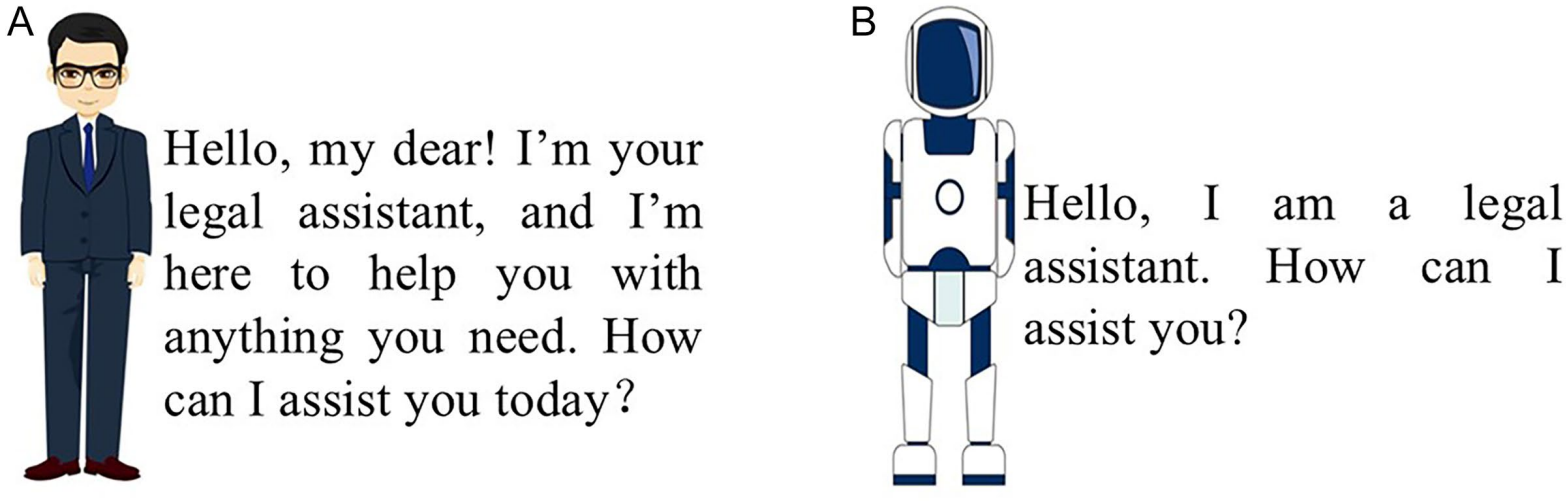
Manipulation of interactional anthropomorphism in Experiment 1. **(A)** High-interactional-anthropomorphism. **(B)** Low-interactional-anthropomorphism.

Pretest 1 was conducted to guarantee the effectiveness of interactional anthropomorphism. The pretest utilized two figures that differed significantly in appearance and communication styles—key dimensions of interactional anthropomorphism—while maintaining identical characteristics in other respects, such as their two-dimensional design and movement postures (see Figures 4A,B). Perception of interactional anthropomorphism was measured using a single question: “To what extent do you think the figure is similar to a human?” (7-point scale, 1 = not at all, 7 = very much). A total of 101 valid responses were collected (51.5% male, M_age = 27.15, SD = 5.69). One-way ANOVA revealed a significantly higher perception of interactional anthropomorphism for the high-interactional-anthropomorphism figure compared to the low-interactional-anthropomorphism figure (M_(high-interactional-anthropomorphism) = 5.29, M_(low-interactional-anthropomorphism) = 2.43, *t* = 13.35, *p* < 0.001). Thus, the experimental material met the control requirements.

#### 5.2.2 Design and participants

Experiment 1 adopted a 2 × 2 between-subjects design to examine the joint effects of dual anthropomorphism, manipulating functional anthropomorphism (successful vs. failed) and interactional anthropomorphism (high vs. low). A total of 291 participants expressing interest in the study were randomly assigned to one of the four groups. After applying an attention check requiring participants to correctly respond to an error-prone number task, 284 valid responses were retained (55.6% male, M_age = 28.52, SD = 7.64, 71 subjects per group).

#### 5.2.3 Procedure and measures

Participants were first presented with the following scenario about pet injury: *“Suppose your pet dog accidentally bites a stranger. After initially negotiating and paying thousands of yuan for medical expenses, the stranger continues to request compensation for additional costs, including mental anguish. To prevent excessive claims, you want to seek information on the specific legal provisions for compensation. Consequently, you visit the community’s law popularization hall and activate the GAI legal service system to inquire about the issue.”*

**Figure 5:**
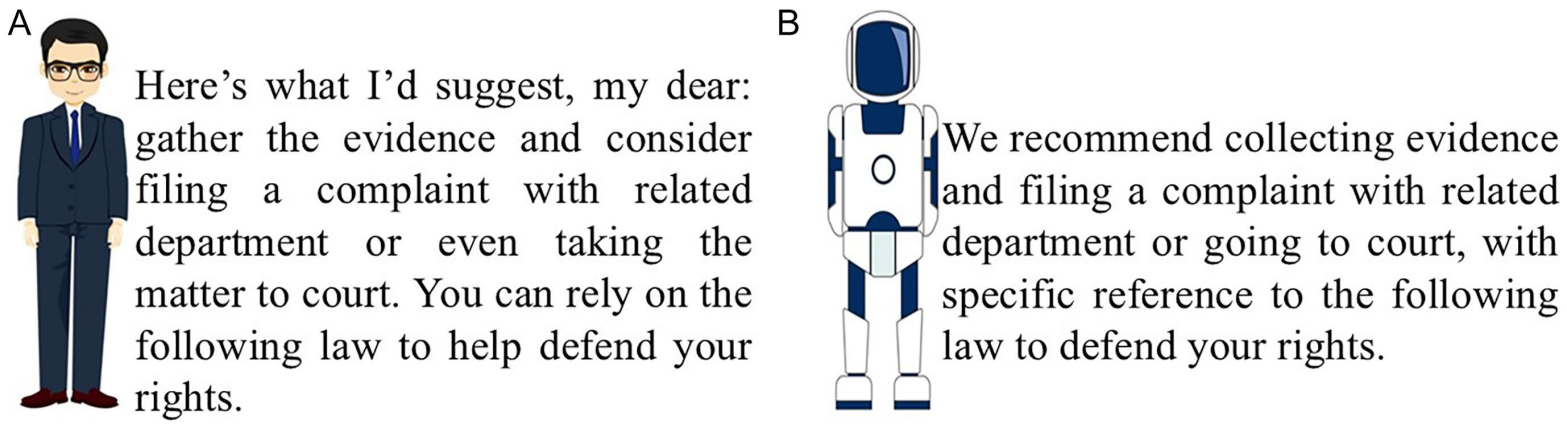
Manipulation of interactional anthropomorphism in successful functional anthropomorphism in Experiment 1 (success). **(A)** High-interactional-anthropomorphism. **(B)** Low-interactional-anthropomorphism.

**Figure 6:**
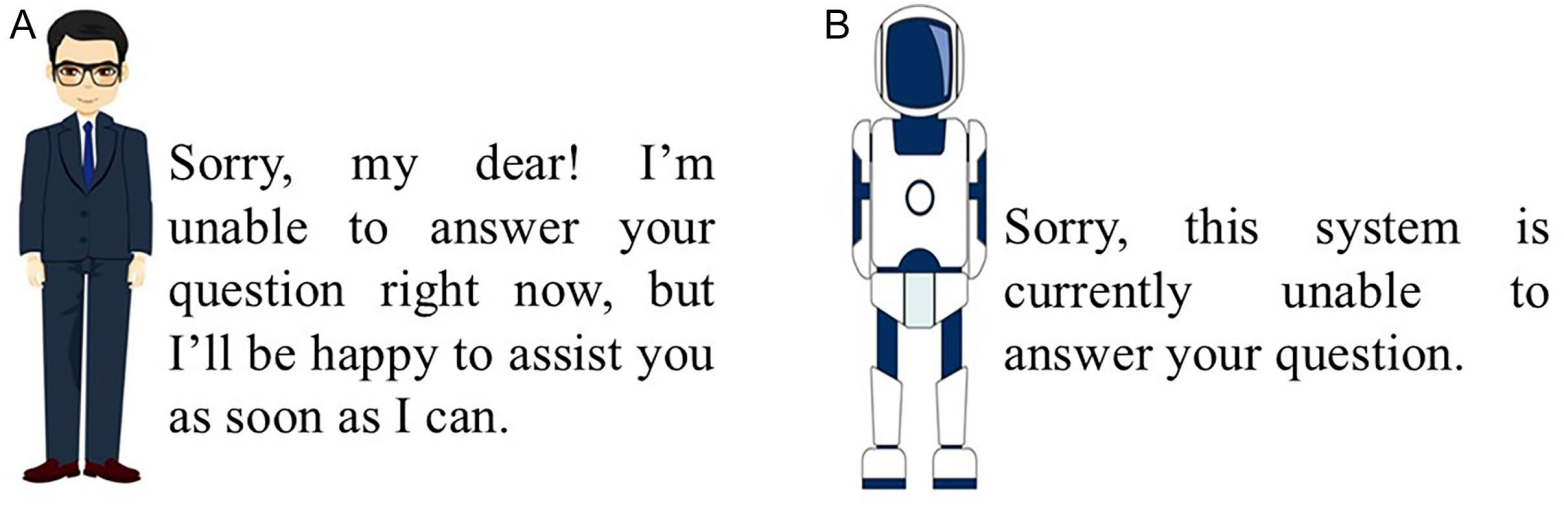
Manipulation of interactional anthropomorphism in failed functional anthropomorphism in Experiment 1 (result failure). **(A)** High-interactional-anthropomorphism. **(B)** Low-interactional-anthropomorphism.

After this, participants in the high-interactional-anthropomorphism group were shown Figure 4A, while those in the low-interactional-anthropomorphism group saw Figure 4B. Participants were then told that if they input the question *“My dog, Albert, bit someone. How should I compensate for it?”* into the GAI system, the GAI lawyer would provide a corresponding answer. Subsequently, Figures 5A,B, 6A,B were presented to the respective groups.

Finally, participants filled out a three-part questionnaire. The first part assessed participants’ perceptions of interactional anthropomorphism, as in Pretest 1. The second part measured continuance intention, according to Bhattacherjee (2001). The items include “I’ll try the GAI lawyer again, not just abandon it,” “I’ll continue to use GAI lawyer, not calling for anyone’s help,” and “I would not stop using GAI lawyer, even if I could choose to stop” (1 = strongly disagree, 7 = strongly agree). The final section collected demographic and contextual variables, including gender, age, perceived cuteness of the GAI, technological familiarity, affinity for technology interaction, and trust in technology. Detailed measurement scales for these control variables are provided in Appendix C.

#### 5.2.4 Results

**Manipulation checks**. One-way ANOVA showed that the perception of interactional anthropomorphism was significantly higher for the high-interactional-anthropomorphism figure compared to the low-interactional-anthropomorphism figure (M_(high-interactional-anthropomorphism) = 5.42, M_(low-interactional-anthropomorphism) = 3.24, *F*(1, 282) = 121.02, *p* < 0.001), Therefore, the experimental manipulation was successful.

**Hypothesis testing of the joint effects**. Given the potential influence of demographic and contextual factors—such as gender, age, GAI cuteness (Lv et al., 2021), technological familiarity (McDonough III and Barczak, 1992), affinity for technology interaction (*α* = 0.92; Franke et al., 2019), and trust in technology (α = 0.93; Flavián et al., 2006)—these variables were included as covariates in the analysis. A two-way ANOVA revealed significant main effects for both interactional anthropomorphism (*F*(1, 280) = 110.61, *p* < 0.001) and functional anthropomorphism (F(1, 280) = 10.02, *p* = 0.002), as well as a significant joint effect (F(1, 280) = 4.11, *p* = 0.044) on users’ continuance intention (details in Appendix D). Users continuance intention in successful functional anthropomorphism was significantly higher than in failed scenarios (M_(successful-functional-anthropomorphism) = 4.91, M_(failed-functional-anthropomorphism) = 4.31, *F* (1, 282) = 8.97, *p* = 0.003), which confirms H1a. Additionally, users exhibited significantly higher continuance intention toward the GAI exhibiting high interactional anthropomorphism compared to exhibiting low interactional anthropomorphism (M_(high-interactional-anthropomorphism) = 5.47, M_(low-interactional-anthropomorphism) = 3.74, F(1, 282) = 99.21, *p* < 0.001), thereby validating H 1b. In the context of GAI exhibiting high interactional anthropomorphism, users continuance intention was significantly higher in successful functional anthropomorphism than in failed scenarios (M_(successful-functional-anthropomorphism) = 5.68, M_(failed-functional-anthropomorphism) = 5.27, *F*(1, 140) = 4.67, *p* = 0.033), thus confirming H 1c. In summary, all sub-hypotheses under H1 were supported.

#### 5.2.5 Discussion

Experiment 1 confirmed the joint effects of GAI’s dual anthropomorphism. The results showed that users exhibited significantly higher continuance intention toward GAI displaying high interactional anthropomorphism compared to low interactional anthropomorphism. Additionally, users’ continuance intention was higher in successful functional anthropomorphism scenarios than in failed ones. Furthermore, high interactional anthropomorphism exerts an augmented effect in successful functional anthropomorphism and a compensatory effect in failed scenarios. However, some studies suggest that the type of failure may influence these effects (Lv et al., 2021). To control for the impact of failure type, we included Experiment 2 as a robustness check.

### 5.3 Experiment 2: robustness test of the joint effects based on functional failure types

#### 5.3.1 Interactional anthropomorphism stimuli

To complement Experiment 1, a single-factor (interactional anthropomorphism: high vs. low) between-subjects design was employed. A total of 146 participants expressing interest in the study were recruited and randomly assigned to one of the two groups. Following the application of attention check questions similar to those in Experiment 1, 142 valid responses were retained (57.7% male; M_age = 27.98, SD = 6.02; 70–72 subjects per group).

#### 5.3.2 Procedure and measures

**Figure 7:**
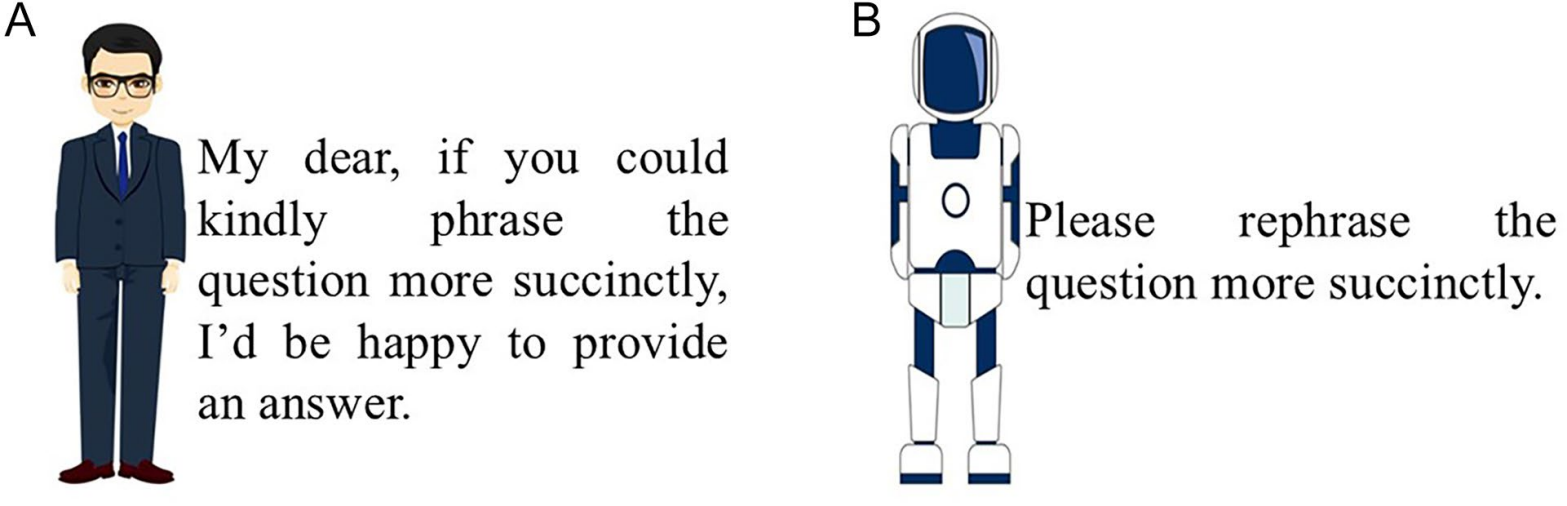
Manipulation of interactional anthropomorphism in failed functional anthropomorphism in Experiment 2 (process failure). **(A)** High-interactional-anthropomorphism. **(B)** Low-interactional-anthropomorphism.

Participants first read the experimental scenario about pet injury described in Experiment 1. Then, the high-interactional-anthropomorphism group was shown Figure 4A, and the low-interactional-anthropomorphism group was shown Figure 4B. Participants were told that if they input the question: *“My pet dog was not supervised properly and bit a stranger. I have already negotiated and settled the medical expenses, amounting to a substantial sum. I believe I have made sufficient compensation, but the man continues to demand additional medical expenses and compensation for emotional distress. How should I handle this to prevent endless claims?”* the GAI lawyer could provide a corresponding answer. Figures 7A,B were then presented to the respective groups.

Next, participants were informed that rephrasing the question in a simpler manner could yield a more useful answer: *“My dog accidentally bit a stranger, and I have compensated for the medical expenses amounting to a substantial sum, but the stranger continues to demand compensation. How should I deal with this issue?”* Following this, Figures 7A,B were shown to the respective groups again.

Subsequently, participants were instructed to use an even simpler phrasing for the question: *“My dog, Albert, bit someone. How should I compensate for it?”* Figures 5A,B were then shown to the respective groups. Afterward, participants completed a questionnaire consistent with Experiment 1.

#### 5.3.3 Results

**Manipulation checks**. One-way ANOVA showed that the perception of interactional anthropomorphism was significantly higher for the high-interactional-anthropomorphism figure than for the low-interactional-anthropomorphism figure (M_(high-interactional-anthropomorphism) = 5.61, M_(low-interactional-anthropomorphism) = 2.85, *F*(1, 140) = 107.50, *p* < 0.001). Thus, the manipulation of interactional anthropomorphism was effective.

**Robustness test of the joint effect**. Data from the two successful functional anthropomorphism groups in Experiment 1 were incorporated to reanalyze the joint effects of dual anthropomorphism. Following the analysis procedure of Experiment 1, all control variables were included in the analysis. The data results were similar to those of Experiment 1, with specific details provided in Appendix E. Therefore, all sub-hypotheses under H1 were re-validated.

**Robustness test of the compensatory effect**. We included data from the two failure groups in Experiment 1 to reanalyze the compensatory effect. As in Experiment 1, all control variables were also included. For continuance intention (*α* = 0.910), two-way ANOVA showed that the main effect of interactional anthropomorphism was significant (*F*(1, 280) = 161.82, *p* < 0.001), the main effect of failure type was not significant (F(1, 280) = 2.25, *p* = 0.135), and the interaction between them was not significant (F(1, 280) = 0.65, *p* = 0.420). These results indicate that, regardless of the failure type of functional anthropomorphism, participants’ intention to continue using GAI was significantly higher when the GAI exhibited high interactional anthropomorphism compared to low interactional anthropomorphism (M_(successful-functional-anthropomorphism) = 5.21, M_(failed-functional-anthropomorphism) = 3.14, *F*(1, 282) = 170.10, *p* < 0.001). Thus, H 1c was further supported.

#### 5.3.4 Discussion

Experiment 2 was conducted as a robustness check for the joint effects of GAI’s dual anthropomorphism identified in Experiment 1. The results confirmed that the type of failure in functional anthropomorphism did not influence the effectiveness of the joint effects. To further explore the underlying mechanism behind the compensatory role of interactional anthropomorphism, Experiment 3 was conducted. In this experiment, a three-dimensional female figure was used to eliminate any potential influences from gender and figure form. Additionally, to enhance the external validity of the study, the background material was modified to include a more common scenario, namely elderly fraud, rather than the previously used context.

### 5.4 Experiment 3: the sequential mechanism of the compensatory effect

#### 5.4.1 Interactional anthropomorphism stimuli

**Figure 8:**
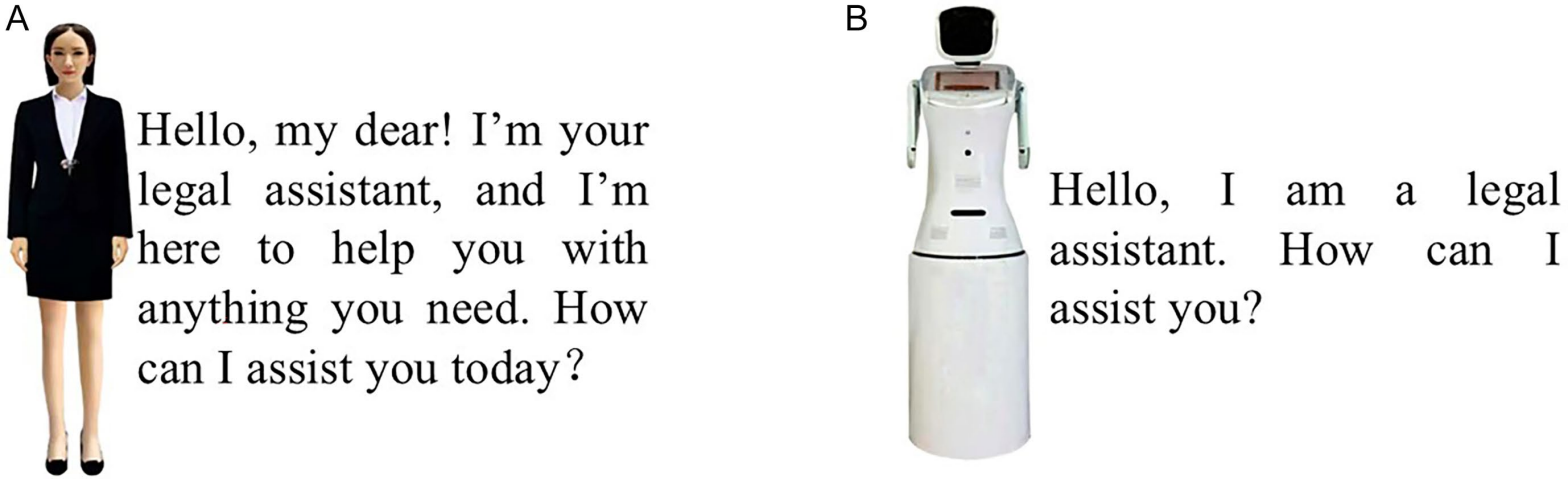
Manipulation of interactional anthropomorphism in Experiment 3. **(A)** High-interactional-anthropomorphism. **(B)** Low-interactional-anthropomorphism.

Pretest 2 was conducted to ensure the effectiveness of interactional anthropomorphism, as in pretest 1. The experimental materials displayed differences in key interactive anthropomorphic features, including appearance and communication styles, while remaining consistent in all other aspects (see Figures 8A,B). A total of 90 valid questionnaires were collected (48.9% male; M_age = 28.43, SD = 4.04). One-way ANOVA showed that the perception of interactional anthropomorphism was significantly higher for the high-interactional-anthropomorphism figure than for the low-interactional-anthropomorphism figure (M_(high-interactional-anthropomorphism) = 5.50, M_(low-interactional-anthropomorphism) = 3.17, *t* = 17.65, *p* < 0.001). Thus, the manipulation was effective.

#### 5.4.2 Design and participants

Experiment 3 employed a single-factor between-subjects design, manipulating interactional anthropomorphism (high vs. low). Since Experiment 2 confirmed that the failure type did not influence the compensatory effect, this experiment specifically focused on result failures to simplify the design. We recruited 180 participants who expressed interest in the study and randomly assigned them to two groups. After applying attention-check questions, as done in Experiment 1, we collected 177 valid responses (male: 48.0%; M_age = 28.44, SD = 6.58; 88–89 subjects per group).

#### 5.4.3 Procedure and measures

Participants first read the experimental materials regarding elderly fraud: *“Suppose your elderly family member attends a health and wellness seminar where the organizers promote a high-end massage device, claiming that its long-term use can extend lifespan and reduce future burdens on children. The elderly person impulsively buys this $1,000 massager, but later discovers that it is an ordinary massager worth only $100. When you contact the seminar organizers, they suddenly stop responding. You want to resolve this issue through legal means, so you visit the community’s legal assistance center and activate the GAI service system for consultation.”*

**Figure 9:**
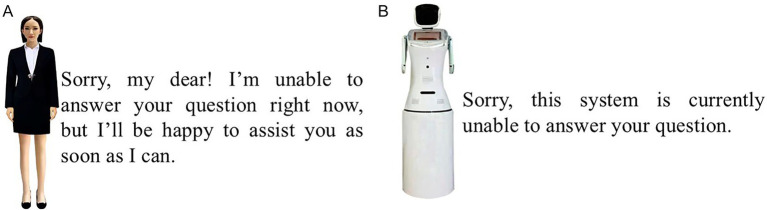
Manipulation of interactional anthropomorphism in Experiment 3. **(A)** High-interactional-anthropomorphism. **(B)** Low-interactional-anthropomorphism.

Next, participants in the high-interactional-anthropomorphism group were shown Figure 8A, and those in the low-interactional-anthropomorphism group were shown Figure 8B. They were then informed that if they asked the GAI lawyer, *“My elderly relative was misled by a health organization into purchasing a severely overpriced massage device. I want to assert my rights, but I am currently unable to contact the relevant representatives. What should I do in this situation?”* the GAI lawyer would provide a corresponding answer. Afterward, Figures 9A,B were shown to the respective groups.

**Figure 10:**
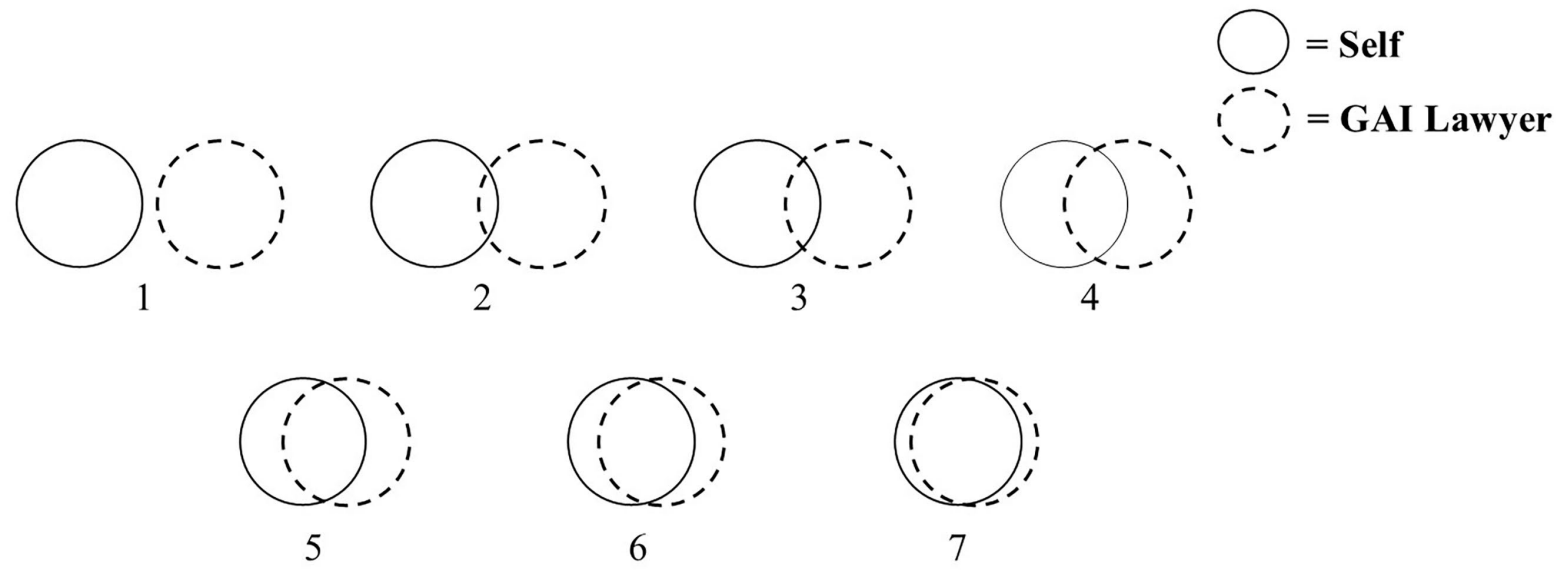
The coincidence scale between self and GAI lawyer.

Finally, participants were invited to complete the questionnaire divided into four parts. The first part measured participants’ perceptions of interactional anthropomorphism, as done in Pretest 1. The second part measured their psychological distance and trust toward the GAI lawyer. The psychological distance measurement drew on the coincidence scale between self and others developed by Pipp et al. (1985) and was adapted to fit the context of this study. Participants were shown Figure 10 and asked, “How do you feel about your relationship with this GAI lawyer?” They selected a picture number indicating the closeness of their relationship with the GAI lawyer (1 = not at all, 7 = very much). Trust in the GAI lawyer was measured with four items on a 7-point scale (1 = strongly disagree, 7 = strongly agree): “I believe this GAI lawyer has the necessary capabilities to solve problems encountered in future services,” “I believe this GAI lawyer has enough experience to solve problems encountered in future services,” “I believe this GAI lawyer has the necessary resources to solve the problems encountered in the service in the future,” and “I believe the big data behind this GAI lawyer understands the problems encountered by users very well and can provide users with the services they need in the future” (Flavián et al., 2006). The third part measured the participants’ continuance intentions, as in Experiment 1. The fourth part assessed some control variables, as in Experiment 1.

#### 5.4.4 Results

**Manipulation checks**. One-way ANOVA showed that the perception of interactional anthropomorphism was significantly higher for the high-interactional-anthropomorphism figure than for the low-interactional-anthropomorphism figure (M_(high-interactional-anthropomorphism) = 5.55, M_(low-interactional-anthropomorphism) = 2.64, *F*(1,175) = 258.51, *p* < 0.001). Thus, the manipulation was successful.

**Hypothesis testing of the joint effects**. One-way ANOVA showed that users had significantly higher continuance intention when the GAI exhibited high interactional anthropomorphism compared to low interactional anthropomorphism (M_(high-interactional-anthropomorphism) = 4.89, M_(low-interactional-anthropomorphism) = 2.88, F(1,175) = 130.30, *p* < 0.001). Thus, this result reverified the compensatory effect of interactional anthropomorphism in H 1.

**Hypothesis testing of mediating effect**. A bootstrap sequential mediation analysis (PROCESS, model 6, 5,000 samples, confidence interval 95%) was used to test the mediating effect (Hayes et al., 2017). The results indicated that the independent mediating effect of psychological distance (*β* = −0.087, LLCI = −0.334, ULCI = 0.171, including 0) was not significant, while the independent mediating effect of trust (*β* = −0.331, LLCI = −0.581, ULCI = −0.143, not including 0) was significant. Furthermore, the sequential mediating effect of psychological distance and trust was significant (*β* = −0.296, LLCI = −0.475, ULCI = −0.147, not including 0). After controlling for these mediators, the effect of interactional anthropomorphism on continuance intention remained significant (*β* = −0.961, LLCI = −1.383, ULCI = −0.539, not including 0), confirming that psychological distance and trust jointly played a partial sequential mediating role. However, reversing the order of the two mediators did not result in a significant sequential mediating effect (*β* = −0.042, LLCI = −0.160, ULCI = 0.085, including 0). Thus, H2 was supported.

#### 5.4.5 Discussion

Experiment 3 not only reverified the compensatory effect of interactional anthropomorphism but also demonstrated that this effect was sequentially mediated by psychological distance and trust. Experiment 4 aimed to examine the moderating effect of task importance. Additionally, to enhance external validity, we presented GAI responses on mobile phone screens within the context of medical disputes and shopping disputes.

### 5.5 Experiment 4: the boundary of the compensatory effect

#### 5.5.1 Task importance stimuli

**Figure 11:**
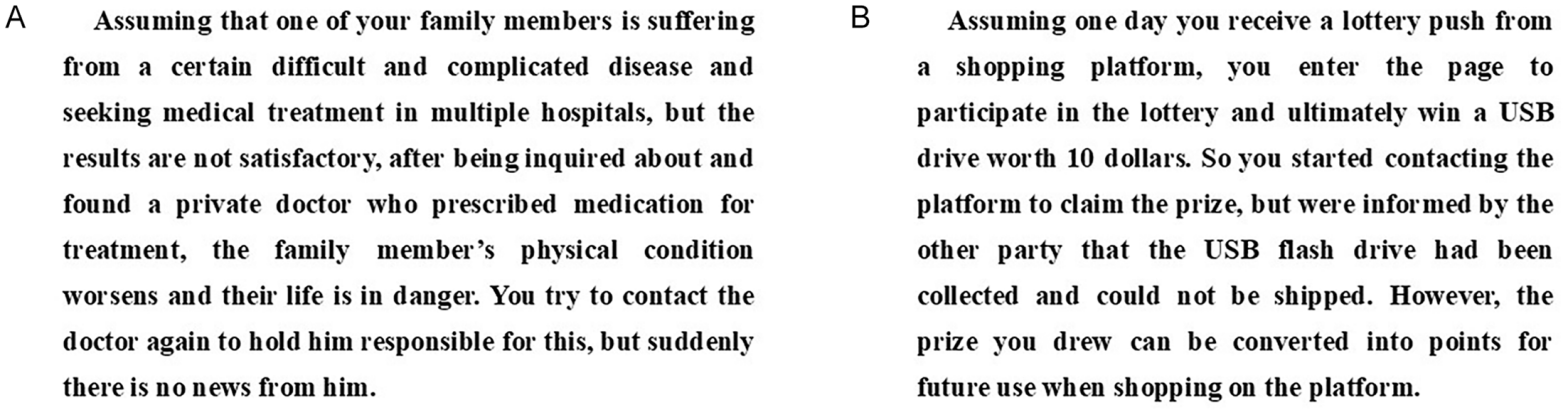
Manipulation of task importance in pretest 3. **(A)** Task importance-high. **(B)** Task importance-low.

The pretest used two scenario materials revolving around medical disputes and shopping disputes, both of which are common and publicly relevant legal issues. These scenarios were designed to represent issues of varying importance: the medical dispute scenario represents a high task importance condition, as it involves health and potential life consequences; the shopping dispute scenario represents a low task importance condition, as it concerns relatively minor financial losses (Figures 11A,B). Participants read a corresponding paragraph describing the task that a GAI lawyer was required to complete (Figures 11A,B), followed by the definition of task importance. Then, participants were asked to rate the level of task importance on a 7-point Likert scale (1 = not important, 7 = very important). We received 94 valid questionnaires (50.0% male; M_age = 28.49, SD = 5.32). The results showed that task importance was perceived as significantly higher in the high-task importance group than in the low-task importance group (M_(high-task importance) = 5.37, M_(low-task importance) = 3.71, *t* = 14.34, *p* < 0.001). Thus, the experimental materials met the manipulation requirements.

#### 5.5.2 Design and participants

Experiment 4 adopted a 2 (interactional anthropomorphism: high vs. low) × 2 (task importance: high vs. low) between-subject design. We recruited 328 participants interested in the experiment and randomly assigned them to one of four experimental groups. Based on the results of attention detection questions as in Experiment 1, a total of 319 questionnaires were returned (48.3% female; M_age = 28.90, SD = 6.22; 79 to 81 subjects per group).

#### 5.5.3 Procedure and measures

**Figure 12:**
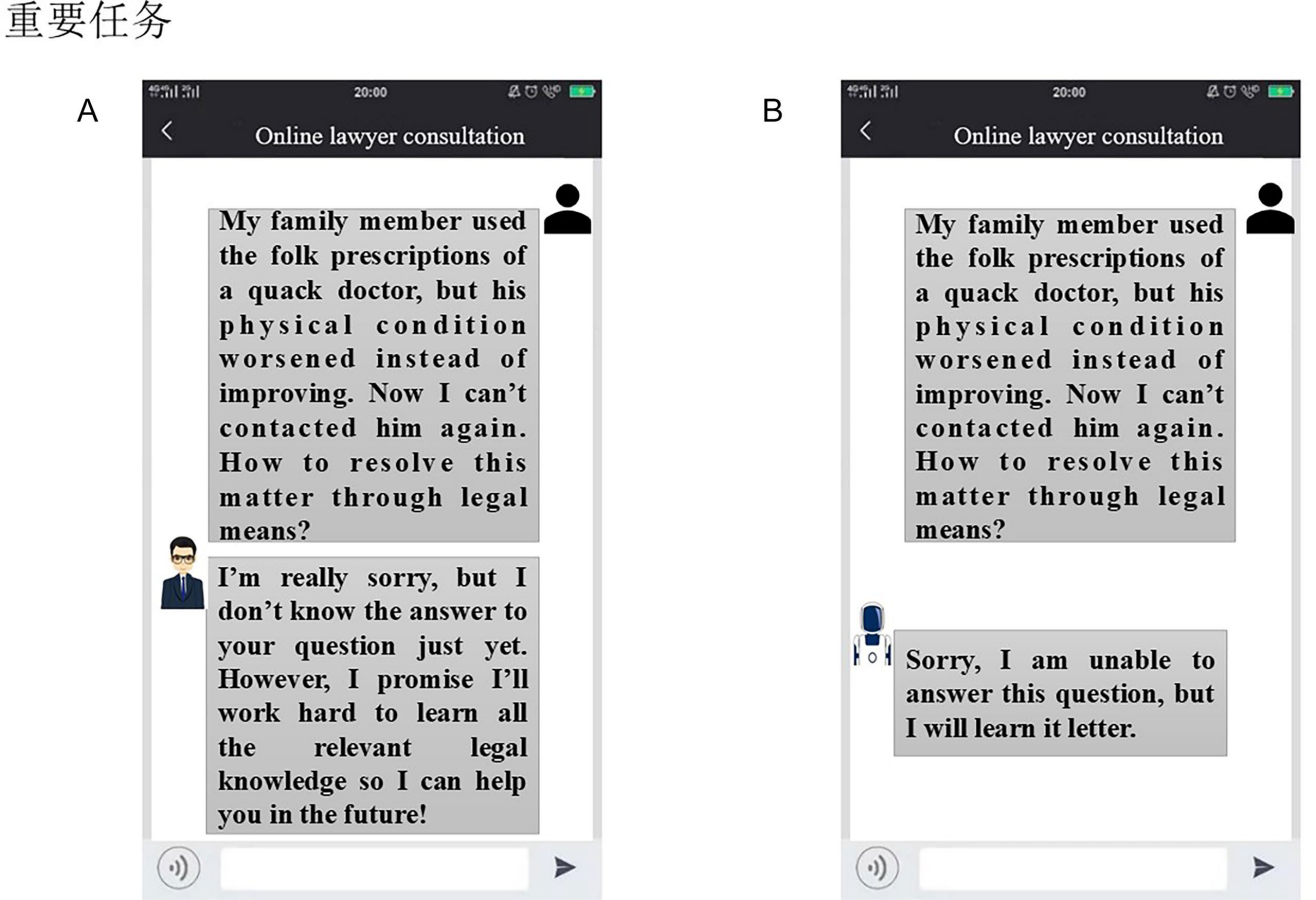
Manipulation of interactional anthropomorphism in Experiment 4 (task importance- high). **(A)** High-interactional-anthropomorphism. **(B)** Low-interactional-anthropomorphism.

**Figure 13:**
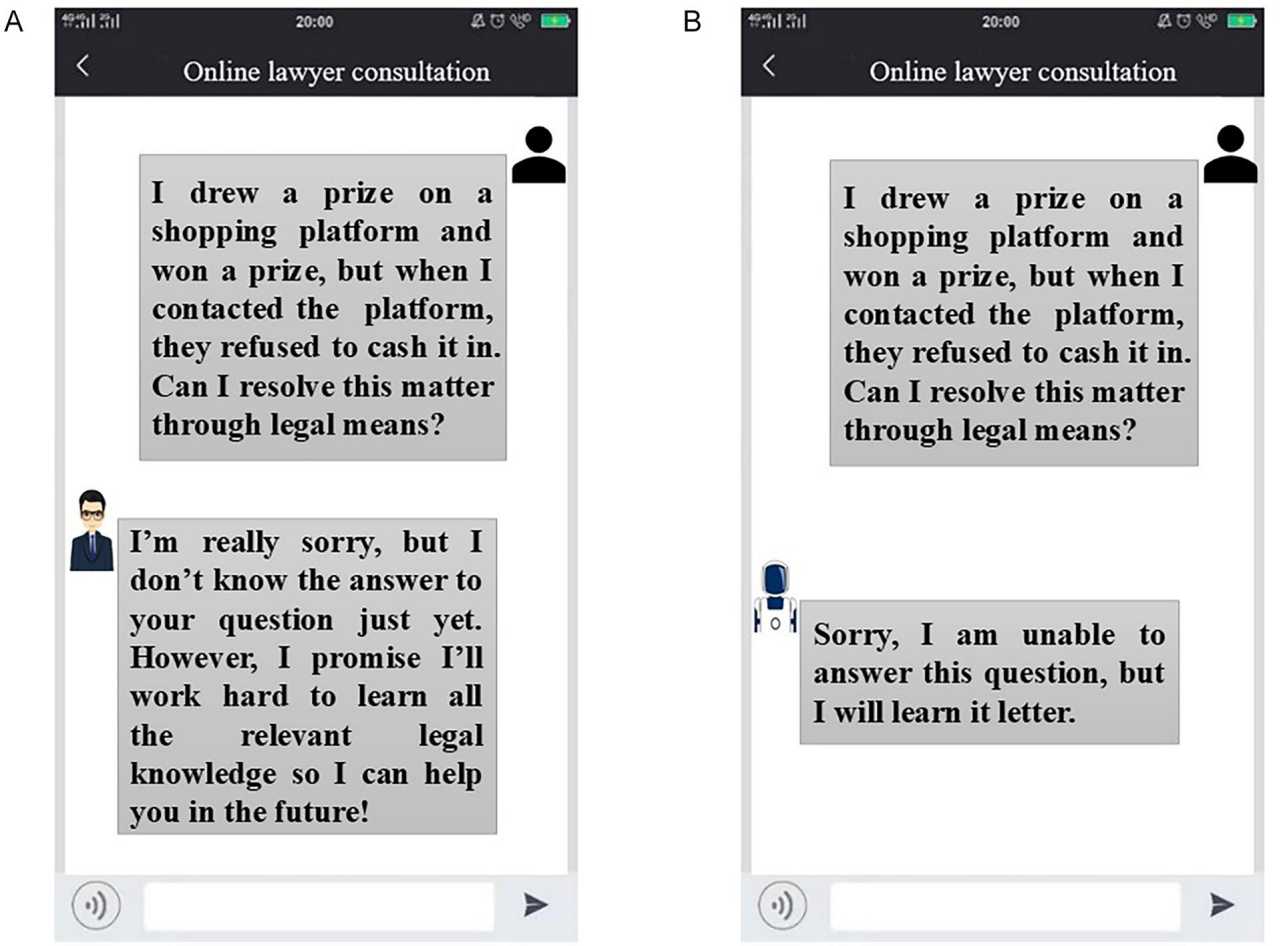
Manipulation of interactional anthropomorphism in Experiment 4 (task importance-low). **(A)** High-interactional-anthropomorphism. **(B)** Low-interactional-anthropomorphism.

Participants were first asked to read the relevant task materials, with the high-task-importance groups reading Figure 11A and the low-task-importance groups reading Figure 11B. They were then informed that the issue could be resolved by a GAI lawyer. Figures 12A,B, 13A,B were presented to the respective groups, showing the manipulation of interactional anthropomorphism. Afterward, participants completed a questionnaire.

The questionnaire consisted of four parts. The first part measured participants’ perceptions of interactional anthropomorphism (consistent with Pretest 1) and task importance (consistent with Pretest 3). The second part assessed psychological distance and trust, as in Experiment 3. The third part measured participants’ continuance intention, as in Experiment 1. The fourth part assessed several control variables, as in Experiment 1.

#### 5.5.4 Results

**Manipulation checks**. Two-way ANOVA showed that for the perception of interactional anthropomorphism, the main effect of interactional anthropomorphism was significant (M_(high-interactional-anthropomorphism) = 5.33, M_(low-interactional-anthropomorphism) = 3.33, *F* (1,315) = 306.59, *p* < 0.001), while the main effect of task importance was not significant (M_(high-task importance) = 4.36, M_(low-task importance) = 4.30, *F*(1,315) = 0.54, *p* = 0.464). The joint effect between interactional anthropomorphism and task importance was not significant (F(1,315) = 0.59, *p* = 0. 442). For the perception of task importance, the main effect of task importance was significant (M_(high-task importance) = 5.13, M_(low-task importance) = 4.60, *F*(1, 315) = 30.60, *p* < 0.001), while the main effect of interactional anthropomorphism was not significant (M_(high-interactional-anthropomorphism) = 4.93, M_(low-interactional-anthropomorphism) = 4.79, F (1,315) = 2.28, *p* = 0.132). The joint effect between them was also not significant (F (1,315) = 0.38, *p* = 0.537). Thus, the experimental manipulation was successful.

**Hypothesis testing of the bounded compensatory effect**. Two-way ANOVA showed that for continuance intention (*α* = 0.888), after controlling all the control variables, the main effect of interactional anthropomorphism was significant (M_(high-interactional-anthropomorphism) = 4.70, M_(low-interactional-anthropomorphism) = 3.95, F(1,315) = 24.10, *p* < 0.001), as well as task importance (M_(high-task importance) = 4.06, M_(low-task importance) = 4.60, F(1,315) = 13.42, *p* < 0.001). Furthermore, the joint effect between them was also significant (F (1,315) = 16.64, *p* < 0.001). Specifically, when task importance was low, users in the high-interactional-anthropomorphism group exhibited significantly higher continuance intention than those in the low-interactional-anthropomorphism group (M_(high-interactional-anthropomorphism) = 5.25, M_(low-interactional-anthropomorphism) = 3.92, *F*(1,158) = 57.66, *p* < 0.001). However, when task importance was high, there was no significant difference in continuance intention between the two groups (M_(high-interactional-anthropomorphism) = 4.14, M_(low-interactional-anthropomorphism) = 3.98, *F*(1,157) = 0.54, *p* = 0. 464; see Appendix F).

**Sequential mediating test**. A Bootstrap method was used to examine the sequentially mediated effect between psychological distance and trust (PROCESS, Model 83, sample size 5,000, confidence interval 95%; Hayes, 2018). The results showed that when task importance was high, the sequential mediating effect of psychological distance and trust was not significant (*β* = −0.077, LLCI = −0.229, ULCI = 0.064, including 0). However, when task importance was low, the sequential mediating effect was significant (*β* = −0.443, LLCI = −0.635, ULCI = −0.284, not excluding 0). After controlling for the mediating variables, the main effect of interactional anthropomorphism on continuance intention remained significant (*β* = −0.256, LLCI = −0.494, ULCI = −0.018, including 0), indicating that psychological distance and trust played a partial mediating role. Thus, H3 was supported.

#### 5.5.5 Discussion

The results of Experiment 4 re-evaluated the compensatory impact of interactional anthropomorphism and test the role of task importance in this compensatory effect. The findings indicated that the compensatory effect of interactional anthropomorphism was more pronounced when the level of task importance was low, whereas the effect was less significant when the task importance was high. The modification of experimental scenarios enhanced the robustness of the research findings.

## 6 Discussion

This study adopts a sequential mixed-method approach to systematically examine the joint effects of functional anthropomorphism and interactional anthropomorphism of generative AI (GAI) on users’ continuance intention. The qualitative phase, based on in-depth interviews, uncovers the underlying mechanisms and boundary conditions of this dual anthropomorphism, laying the foundation for the subsequent hypothesis development. The quantitative phase then empirically tests these mechanisms through experimental validation. The integration of the two phases not only enriches our understanding of how GAI anthropomorphism influences user behavior but also offers practical insights for designing and optimizing human–GAI interaction systems.

Our findings reveal that the interplay between functional and interactional anthropomorphism exerts a significant influence on continuance intention. When functional anthropomorphism performs well, users’ continuance intention increases markedly. Under such conditions, high levels of interactional anthropomorphism exert an amplifying effect, further enhancing satisfaction and trust. However, when functional anthropomorphism fails, interactional anthropomorphism demonstrates a compensatory effect—mitigating users’ disappointment through affective resonance and relational warmth, thereby sustaining a relatively positive attitude toward use. Further analysis suggests that this compensatory effect operates primarily through reducing psychological distance and restoring trust. Moreover, task importance serves as a critical boundary condition: when task importance is low, the compensatory role of interactional anthropomorphism becomes more salient, whereas in high-importance tasks, users prioritize instrumental competence over emotional connection, reducing the space for affective compensation.

At the theoretical level, this study extends the Expectation Confirmation Model (ECM) by integrating the dual dimensions of anthropomorphism to explain how distinct types of human-likeness jointly shape users’ confirmation and evaluation processes. Traditional ECM focuses primarily on the cognitive consistency between user expectations and system performance, often overlooking the relational and emotional dimensions of human–AI interaction. Our results show that functional and interactional anthropomorphism contribute differently yet complementarily to the ECM process. Functional anthropomorphism primarily relates to cognitive expectations—before interaction, it helps users form judgments about whether the GAI system can fulfill their task requirements by demonstrating logical reasoning and problem-solving ability. During interaction, the system’s actual performance confirms or disconfirms these expectations, influencing users’ satisfaction at the cognitive level. In contrast, interactional anthropomorphism mainly operates in the affective and relational reappraisal that follows interaction. When cognitive expectations are unmet, socially responsive and empathic behaviors enable users to reinterpret their disappointment, reducing dissatisfaction by restoring trust and relational warmth. When expectations are met, interactional anthropomorphism further amplifies positive affect, reinforcing satisfaction and continuance intention.

This dynamic coupling highlights a dual-path mechanism within the ECM: functional anthropomorphism drives a cognitive confirmation path, whereas interactional anthropomorphism drives an affective reappraisal path. Together, they shape users’ overall evaluations across different stages of interaction—users’ continuance decisions thus depend not only on whether the system “performs well” but also on whether it “responds like a human.” By embedding dual anthropomorphism into the ECM, this study extends the model from a performance-centered framework to a more holistic structure that captures both cognitive and emotional processes in user evaluations of GAI. This theoretical integration enhances the explanatory power of ECM and offers a more nuanced understanding of human–AI relationships. It reveals that in the era of generative AI, users’ continuance intentions are no longer driven solely by assessments of functional reliability, but also deeply rooted in the trust, empathy, and relational experiences that emerge during interaction.

### 6.1 Theoretical contribution

This article makes three significant theoretical contributions, advancing the understanding of GAI anthropomorphism and its impacts. First, it deepens the understanding of the anthropomorphic characteristics of GAI—an increasingly prominent technological feature in the current development of GAI. While prior studies in social robotics and chatbot design have discussed functional and social (or interactional) anthropomorphism separately, the present research advances the literature by recontextualizing these dimensions in the generative AI (GAI) domain, where anthropomorphic cues are not pre-programmed but emerge dynamically from generative capabilities. Unlike rule-based chatbots or embodied robots, GAI simultaneously performs high-level reasoning (functional anthropomorphism) and expressive communication (interactional anthropomorphism) in multimodal contexts. This study thus contributes a dual-dimensional framework that treats functional and interactional anthropomorphism as interdependent and co-evolving rather than parallel traits. The framework reveals how these two dimensions jointly produce augmented and compensatory effects—outcomes that have not been theorized in previous anthropomorphism literature. This theoretical integration underscores why GAI represents a qualitatively new context for anthropomorphism research and yields fresh insights into human–AI interaction.

Second, this study advances theoretical integration by extending the Expectation Confirmation Model (ECM) to incorporate the dual anthropomorphism of generative AI. Traditional ECM frameworks emphasize cognitive alignment between user expectations and system performance but often overlook the emotional and relational dynamics in human-like interactions. Our findings show that functional anthropomorphism shapes users’ initial cognitive evaluations of whether GAI meets task-oriented expectations, whereas interactional anthropomorphism influences post-experience affective evaluations by fostering trust and reducing psychological distance. Integrating these two dimensions transforms ECM into a dual-path framework that captures both instrumental and relational evaluations of GAI, offering a more comprehensive explanation of how cognitive and emotional confirmations jointly drive continuance intention.

Third, our research responds to the current call in the IS field for user-centered research by shifting the focus from the technological features embodied by GAI to the impact of these features on users’ expectations and experiences. With GAI increasingly forming partner-like relationships with users, the duality of expectations—task-oriented cognitive needs and interaction-driven emotional needs—has become more pronounced. However, existing studies have primarily focused on cognitive experiences, often overlooking the emotional dimensions of HGAII. Our research emphasizes the interaction between technological design and the multidimensional nature of user needs, offering a nuanced understanding of the complex mechanisms and relationships that underpin these effects. In doing so, it addresses the growing call for user-centered research in IS.

### 6.2 Practical contribution

This study offers several actionable implications for the design, deployment, and management of generative AI (GAI) systems. From a human-centered perspective, the findings emphasize that integrating both cognitive and emotional needs into GAI development is essential to improving user experience and fostering sustainable engagement. Designers should therefore enhance functional and interactional anthropomorphism in a coordinated manner, ensuring that the system not only performs tasks accurately but also communicates in a way that feels socially and emotionally intelligent.

First, GAI developers should differentiate their anthropomorphic design strategies based on task importance. For high-importance tasks—such as medical advice, financial decision support, or professional analysis—users prioritize cognitive reliability. Designers should focus on strengthening functional anthropomorphism, such as improving contextual reasoning, factual accuracy, and adaptive problem-solving. In these contexts, interactional anthropomorphism should play a supporting role: a calm, professional, and confidence-inducing communication style can reinforce trust without distracting from task competence. For low-importance or emotionally engaging tasks—such as creative writing, fitness coaching, or lifestyle assistance—users place greater value on emotional connection. Here, developers should emphasize interactional anthropomorphism, incorporating warmth, empathy, humor, and personalization into voice, text, and visual interfaces to deepen engagement.

Second, the results underscore the importance of trust calibration mechanisms in GAI system design. When functional failures occur, interactional anthropomorphism can serve as a compensatory mechanism by reducing psychological distance and sustaining trust. Developers can operationalize this insight through features such as adaptive apology templates, transparent error explanations, or empathic re-engagement prompts that humanize system limitations while maintaining credibility.

Third, from a user interface (UI) perspective, designers should integrate anthropomorphic cues strategically rather than uniformly. For instance, in text-based interfaces, adaptive tone modulation and personalized linguistic style can strengthen interactional anthropomorphism. In multimodal environments (e.g., avatars or voice agents), designers can use subtle nonverbal cues—eye contact, facial micro-expressions, or prosodic variation—to evoke social presence without overwhelming the cognitive dimension. These strategies should be dynamically tuned according to user profiles and task contexts.

Fourth, GAI deployers should implement personalized interaction management and context monitoring mechanisms. The findings show that the effectiveness of interactional anthropomorphism depends on task importance; thus, organizations can adopt adaptive algorithms that adjust interaction style based on user engagement level and situational urgency. In low-importance tasks, higher expressiveness and affective warmth can sustain user satisfaction, while in high-importance tasks, minimizing unnecessary anthropomorphic cues and emphasizing accuracy can prevent user frustration. Such adaptive management not only enhances satisfaction but also mitigates customer churn risk when functional shortcomings occur.

Finally, these insights highlight that successful GAI deployment requires a balance between technological precision and emotional intelligence. By aligning the dual dimensions of anthropomorphism with task characteristics, organizations can design AI systems that are both competent partners and empathetic companions, ultimately fostering trust, satisfaction, and long-term engagement.

### 6.3 Limitations and future directions

This research has several limitations. First, the experimental materials relied on static avatar images and scripted interaction sequences to ensure control and comparability across conditions. While this design enhances internal validity, it simplifies the temporal and adaptive nature of real human–GAI communication. Future studies should employ dynamic and interactive designs that allow participants to engage with generative systems in real time, capturing emotional fluctuations, adaptation strategies, and evolving trust dynamics. Longitudinal studies tracking user perceptions over extended periods could further reveal how functional and interactional anthropomorphism jointly influence sustained engagement and relational bonding. Such approaches would provide richer, temporally grounded insights into human–GAI co-adaptation processes.

Second, the experimental scenarios were limited to legal consultation tasks using a ChatGPT-like system with visual avatars. While this context effectively captured users’ cognitive and emotional expectations, it constrains ecological validity. Anthropomorphic effects may vary across domains such as education, creativity, and mental health, where users’ goals, emotional involvement, and task significance differ. Future studies should therefore extend this framework to diverse GAI contexts and compare how contextual differences shape the compensatory and augmented effects of dual anthropomorphism.

Third, the manipulation of functional anthropomorphism in this study was simplified to represent one-time failures, aligning with common practices in empirical research on service failures. While this approach facilitated the experimental design, it restricted the exploration of the effects of interactional anthropomorphism in ongoing or complex failure scenarios. Future research should address this limitation by examining the role of interactional anthropomorphism in mitigating the impact of recurring or multifaceted functional failures to provide a more nuanced understanding of its compensatory effects.

Fourth, the exploration of boundary conditions for anthropomorphism’s effects requires further investigation. While task importance emerged as a critical boundary in our qualitative research and was the primary focus of this study, other potential boundaries warrant further investigation, such as characteristics of failure, other characteristics of the task, technical features (see Appendix A) Future studies should incorporate these factors to refine the proposed extended ECM and offer a more comprehensive framework for understanding the effects of GAI anthropomorphism.

Finally, despite our rigorous approach, our qualitative analysis contained some limitations. The qualitative sample size, while sufficient for theoretical saturation, limits generalizability across broader populations. Moreover, although reflexivity logs, member checking, and external review minimized researcher bias, the analytical interpretations may still reflect our disciplinary perspectives. Future research could benefit from incorporating interdisciplinary coding teams, cross-cultural samples, and longitudinal observations of naturalistic GAI use to further enhance robustness and validity.

## 7 Conclusion

This research employs a mixed-methods approach to examine the joint effects of GAI’s dual anthropomorphism. With advancements in LLMs and multimodal technologies, GAI anthropomorphism can be categorized into functional anthropomorphism and interactional anthropomorphism. This research proposes an extended ECM to offer a more comprehensive understanding of its joint effect. The conclusions are organized into three parts. The first part verifies the joint effects of dual anthropomorphism. Additionally, the joint effect implies that high interactional anthropomorphism exerts an augmented effect in successful functional anthropomorphism and a compensatory effect in failed scenarios. The second part verifies the sequential mediating roles of psychological distance and trust in the compensatory effect of interactional anthropomorphism. The third part assesses the moderating role of task importance in the compensatory effect, demonstrating that the effect is more pronounced for low-importance tasks than for high-importance tasks. This research also offers important guidance for practice.

## Data Availability

The raw data supporting the conclusions of this article will be made available by the authors, without undue reservation.

## References

[ref1] Alavi M. Leidner D. Mousavi R. (2024). A knowledge management perspective of generative artificial intelligence. J. Assoc. Inf. Syst. 25, 1–12. doi: 10.17705/1jais.00859

[ref2] Anderson R. E. (1973). Consumer dissatisfaction: the effect of disconfirmed expectancy on perceived product performance. J. Mark. Res. 10, 38–44.

[ref3] Anderson E. W. Sullivan M. W. (1993). The antecedents and consequences of customer satisfaction for firms. Mark. Sci. 12, 125–143.

[ref4] Bhattacherjee A. (2001). Understanding information systems continuance: an expectation-confirmation model. MIS Q. 25, 351–370. doi: 10.2307/3250921

[ref5] Blut M. Wang C. Wünderlich N. V. Brock C. (2021). Understanding anthropomorphism in service provision: a meta-analysis of physical robots, chatbots, and other AI. J. Acad. Mark. Sci. 49, 632–658. doi: 10.1007/s11747-020-00762-y, PMID: 41159878

[ref6] Bonneviot F. Coeugnet S. Brangier E. (2023). How to improve pedestrians' trust in automated vehicles: new road infrastructure, external human–machine interface with anthropomorphism, or conventional road signaling? Front. Psychol. 14:1129341. doi: 10.3389/fpsyg.2023.1129341, PMID: 37213373 PMC10196377

[ref7] Brendel A. B. Hildebrandt F. Dennis A. R. Riquel J. (2023). The paradoxical role of humanness in aggression toward conversational agents. J. Manag. Inf. Syst. 40, 883–913. doi: 10.1080/07421222.2023.2229127

[ref8] Brown S. A. Venkatesh V. Goyal S. (2014). Expectation confirmation in information systems research. MIS Q. 38, 729–756. doi: 10.25300/MISQ/2014/38.3.05

[ref9] Chakraborty D. Kar A. K. Patre S. Gupta S. (2024). Enhancing trust in online grocery shopping through generative AI chatbots. J. Bus. Res. 180:114737. doi: 10.1016/j.jbusres.2024.114737

[ref10] Chen J. Li M. Ham J. (2024). Different dimensions of anthropomorphic design cues: how visual appearance and conversational style influence users’ information disclosure tendency towards chatbots. Int. J. Human-Computer Stud. 190:103320. doi: 10.1016/j.ijhcs.2024.103320, PMID: 41159119

[ref11] Chuanyang H. He Q. (2025). Enhancing memory retrieval in generative agents through LLM-trained cross attention networks. Front. Psychol. 16:1591618. doi: 10.3389/fpsyg.2025.1591618 40400752 PMC12092450

[ref12] Churchill G. A. Jr. Surprenant C. (1982). An investigation into the determinants of customer satisfaction. J. Mark. Res. 19, 491–504.

[ref13] Cropanzano R. (2005). Social exchange theory: an interdisciplinary review. Aust. J. Manag. 31, 874–900. doi: 10.1177/0149206305279602

[ref14] Dang J. Liu L. (2023). Do lonely people seek robot companionship? A comparative examination of the loneliness–robot anthropomorphism link in the United States and China. Comput. Hum. Behav. 141:107637. doi: 10.1016/j.chb.2022.107637

[ref15] Dwivedi Y. K. Kshetri N. Hughes L. Slade E. L. Jeyaraj A. Kar A. K. . (2023). Opinion paper: “so what if ChatGPT wrote it?” multidisciplinary perspectives on opportunities, challenges and implications of generative conversational AI for research, practice and policy. Int. J. Inf. Manag. 71:102642. doi: 10.1016/j.ijinfomgt.2023.102642

[ref16] Epley N. Waytz A. Cacioppo J. T. (2007). On seeing human: a three-factor theory of anthropomorphism. Psychol. Rev. 114, 864–886. doi: 10.1037/0033-295X.114.4.864, PMID: 17907867

[ref17] Flavián C. Guinalíu M. Gurrea R. (2006). The role played by perceived usability, satisfaction and consumer trust on website loyalty. Inf. Manag. 43, 1–14. doi: 10.1016/j.im.2005.01.002

[ref18] Franke T. Attig C. Wessel D. (2019). A personal resource for technology interaction: development and validation of the affinity for technology interaction (ATI) scale. Int. J. Hum.-Comput. Interact. 35, 456–467. doi: 10.1080/10447318.2018.1456150

[ref19] Fui-Hoon Nah F. Zheng R. Cai J. Siau K. Chen L. (2023). Generative AI and ChatGPT: applications, challenges, and AI-human collaboration. J. Inf. Technol. Case Appl. Res. 25, 277–304. doi: 10.1080/15228053.2023.2233814

[ref20] Gessinger I. Seaborn K. Steeds M. Cowan B. R. (2025). ChatGPT and me: first-time and experienced users’ perceptions of ChatGPT’s communicative ability as a dialogue partner. Int. J. Hum.-Comput. Stud. 194:103400. doi: 10.1016/j.ijhcs.2024.103400

[ref21] Hayes A. F. (2018). Partial, conditional, and moderated moderated mediation: quantification, inference, and interpretation. Commun. Monogr. 85, 4–40. doi: 10.1080/03637751.2017.1352100

[ref22] Hayes A. F. Montoya A. K. Rockwood N. J. (2017). The analysis of mechanisms and their contingencies: PROCESS versus structural equation modeling. Australas. Mark. J. 25, 76–81. doi: 10.1016/j.ausmj.2017.02.001

[ref23] Hua Y. Cheng X. Hou T. Luo R. (2020). Monetary rewards, intrinsic motivators, and work engagement in the IT-enabled sharing economy: a mixed-methods investigation of internet taxi drivers. Decis. Sci. 51, 755–785. doi: 10.1111/deci.12372

[ref24] Johnston R. (1995). The zone of tolerance: exploring the relationship between service transactions and satisfaction with the overall service. Int. J. Serv. Ind. Manag. 6, 46–61.

[ref25] Jussupow E. Spohrer K. Heinzl A. Gawlitza J. (2021). Augmenting medical diagnosis decisions? An investigation into physicians’ decision-making process with artificial intelligence. Inf. Syst. Res. 32, 713–735. doi: 10.1287/isre.2020.0980, PMID: 19642375

[ref26] Kim D. J. Ferrin D. L. Rao H. R. (2009). Trust and satisfaction, two stepping stones for successful e-commerce relationships: a longitudinal exploration. Inf. Syst. Res. 20, 237–257. doi: 10.1287/isre.1080.0188, PMID: 19642375

[ref27] Kim K. Zhang M. Li X. (2008). Effects of temporal and social distance on consumer evaluations. J. Consum. Res. 35, 706–713. doi: 10.1086/592131

[ref28] Kopp T. Baumgartner M. Kinkel S. (2022). How linguistic framing affects factory workers' initial trust in collaborative robots: the interplay between anthropomorphism and technological replacement. Int. J. Hum.-Comput. Stud. 158:102730. doi: 10.1016/j.ijhcs.2021.102730

[ref29] Kshetri N. Dwivedi Y. K. Davenport T. H. Panteli N. (2024). Generative artificial intelligence in marketing: applications, opportunities, challenges, and research agenda. Int. J. Inf. Manag. 75:102716. doi: 10.1016/j.ijinfomgt.2023.102716

[ref30] Küchemann S. Rau M. Schmidt A. Kuhn J. (2024). Chatgpt's quality: reliability and validity of concept inventory items. Front. Psychol. 15:1426209. doi: 10.3389/fpsyg.2024.1426209, PMID: 39439749 PMC11493723

[ref31] Li J. Huang J. Ni B. (2024). Machine communicative responsibility perception: functional and emotional communicative responsibility of AI advisors and AI partners. Int. J. Hum.-Comput. Interact. 40, 4772–4786. doi: 10.1080/10447318.2023.2221603

[ref32] Li X. Sung Y. (2021). Anthropomorphism brings us closer: the mediating role of psychological distance in user–AI assistant interactions. Comput. Hum. Behav. 118:106680. doi: 10.1016/j.chb.2021.106680

[ref33] Liljander V. Strandvik T. (1993). Estimating zones of tolerance in perceived service quality and perceived service value. Int. J. Serv. Ind. Manag. 4, 6–28.

[ref34] Locke E. A. Shaw K. N. Saari L. M. Latham G. P. (1981). Goal setting and task performance: 1969–1980. Psychol. Bull. 90, 125–152.

[ref35] Lu Z. Min Q. Jiang L. Chen Q. (2024). The effect of the anthropomorphic design of chatbots on customer switching intention when the chatbot service fails: an expectation perspective. Int. J. Inf. Manag. 76:102767. doi: 10.1016/j.ijinfomgt.2024.102767

[ref36] Lv X. Liu Y. Luo J. Liu Y. Li C. (2021). Does a cute artificial intelligence assistant soften the blow? The impact of cuteness on customer tolerance of assistant service failure. Ann. Tour. Res. 87:103114. doi: 10.1016/j.annals.2020.103114

[ref37] Lv X. Yang Y. Qin D. Cao X. Xu H. (2022). Artificial intelligence service recovery: the role of empathic response in hospitality customers’ continuous usage intention. Comput. Hum. Behav. 126:106993. doi: 10.1016/j.chb.2021.106993

[ref38] Mattke J. Maier C. Reis L. Weitzel T. (2021). Bitcoin investment: a mixed methods study of investment motivations. Eur. J. Inf. Syst. 30, 261–285. doi: 10.1080/0960085X.2020.1787109

[ref39] McDonough E. F. III Barczak G. (1992). The effects of cognitive problem-solving orientation and technological familiarity on faster new product development. J. Prod. Innov. Manag. 9, 44–52.

[ref40] Nan D. Sun S. Zhang S. Zhao X. Kim J. H. (2025). Analyzing behavioral intentions toward generative artificial intelligence: the case of ChatGPT. Univ. Access Inf. Soc., 24, 885–895. doi: 10.1007/s10209-024-01116-z

[ref41] Nass C. Moon Y. (2000). Machines and mindlessness: social responses to computers. J. Soc. Issues 56, 81–103. doi: 10.1111/0022-4537.00153

[ref42] Oliver R. L. (1980). A cognitive model of the antecedents and consequences of satisfaction decisions. J. Mark. Res. 17, 460–469.

[ref43] Pipp S. Shaver P. Jennings S. Lamborn S. Fischer K. W. (1985). Adolescents’ theories about the development of their relationships with parents. J. Pers. Soc. Psychol. 48, 991–1001. doi: 10.1037/0022-3514.48.4.991, PMID: 3989677

[ref44] Schuetzler R. M. Grimes G. M. Scott Giboney J. (2020). The impact of chatbot conversational skill on engagement and perceived humanness. J. Manag. Inf. Syst. 37, 875–900. doi: 10.1080/07421222.2020.1790204

[ref45] Song Q. Wang Y. Chen Y. Benitez J. Hu J. (2019). Impact of the usage of social media in the workplace on team and employee performance. Inf. Manag. 56:103160. doi: 10.1016/j.im.2019.04.003

[ref46] Spatola N. Chaminade T. (2022). Cognitive load increases anthropomorphism of humanoid robot. The automatic path of anthropomorphism. Int. J. Hum.-Comput. Stud. 167:102884. doi: 10.1016/j.ijhcs.2022.102884

[ref47] Tashakkori A. Teddlie C. (1998). Mixed Methodology: Combining Qualitative and Quantitative Approaches. Thousand Oaks, CA: SAGE..

[ref48] Tian W. Ge J. Zhao Y. Zheng X. (2024). AI Chatbots in Chinese higher education: adoption, perception, and influence among graduate students—an integrated analysis utilizing UTAUT and ECM models. Front. Psychol. 15:1268549. doi: 10.3389/fpsyg.2024.1268549, PMID: 38384353 PMC10879389

[ref49] Ullman S. Samtani S. Zhu H. Lazarine B. Chen H. Nunamaker J. F. Jr. (2024). Enhancing vulnerability prioritization in cloud computing using multi-view representation learning. J. Manag. Inf. Syst. 41, 708–743. doi: 10.1080/07421222.2024.2376384

[ref50] Venkatesh V. Brown S. Bala H. (2013). Bridging the qualitative-quantitative divide: guidelines for conducting mixed methods research in information systems. MIS Q. 37, 21–54. doi: 10.25300/MISQ/2013/37.1.02

[ref51] Venkatesh V. Brown S. Sullivan Y. (2016). Guidelines for conducting mixed-methods research: an extension and illustration. J. Assoc. Inf. Syst. 17, 435–494. doi: 10.17705/1jais.00433

[ref52] Venkatesh V. Goyal S. (2010). Expectation disconfirmation and technology adoption: polynomial modeling and response surface analysis. MIS Q. 34, 281–303. doi: 10.2307/20721428

[ref53] Waytz A. Heafner J. Epley N. (2014). The mind in the machine: anthropomorphism increases trust in an autonomous vehicle. J. Exp. Soc. Psychol. 52, 113–117. doi: 10.1016/j.jesp.2014.01.005

[ref54] Webster C. Sundaram D. S. (2009). Effect of service provider's communication style on customer satisfaction in professional services setting: the moderating role of criticality and service nature. J. Serv. Mark. 23, 103–113. doi: 10.1108/08876040910946369

[ref55] Xin X. Liu W. (2025). Exploring the balance between functionality and aesthetics: an analytical framework and pragmatic consideration of the anthropomorphism of service robots. Front. Psychol. 16:1555395. doi: 10.3389/fpsyg.2025.1555395, PMID: 40242732 PMC12000057

[ref56] Yang X. Song B. Chen L. Ho S. S. Sun J. (2025). Technological optimism surpasses fear of missing out: a multigroup analysis of presumed media influence on generative AI technology adoption across varying levels of technological optimism. Comput. Hum. Behav. 162:108466. doi: 10.1016/j.chb.2024.108466

[ref57] Yao X. Xi Y. (2024). Pathways linking expectations for AI chatbots to loyalty: a moderated mediation analysis. Technol. Soc. 78:102625. doi: 10.1016/j.techsoc.2024.102625

[ref58] Zhou T. Zhang C. (2024). Examining generative AI user addiction from a CAC perspective. Technol. Soc. 78:10265. doi: 10.1016/j.techsoc.2024.102653

