# Supplementary material for: Decoding the duality of GAI anthropomorphism and its joint effects—a sequential mixed-methods approach
Source: Front Psychol. 2025 Nov 28;16:1615342. doi: 10.3389/fpsyg.2025.1615342 (PMC12698472; doi:10.3389/fpsyg.2025.1615342)
Supplement: Supplementary file 1 [file Supplementary_file_1.docx]

**Appendix**

**Appendix A. Interviewee’ demographic information and interactive experiences.**

| Participant | Gender | Age | Occupation | Dual Expectation | Joint effect | Successfully answered questions | Insufficiently  answered questions | Mechanism | Boundary-task importance | Other boundary condition |
| --- | --- | --- | --- | --- | --- | --- | --- | --- | --- | --- |
| P 1 | Female | 23 | Master student | **√** | **√** | The impact of AI on work patterns and workflows | A psychological activity planning proposal | **√** | **√** |  |
| P 2 | Male | 28 | Programmer | **√** | **√** | Python code writing | Hexadecimal conversion | **√** | **√** |  |
| P 3 | Male | 26 | PhD student | **√** | **√** | Precise lunch recommendations | Lunch recommendations | **√** | **√** | Service failure severity |
| P 4 | Male | 26 | Financial analyst | **√** | **√** | Potato cooking methods | Introduction to the history of mathematics | **√** | **√** | Task standardization; frequency of errors |
| P 5 | Female | 24 | PhD student | **√** | **√** | Variety show introduction | Movie recommendations | **√** | **√** |  |
| P 6 | Male | 32 | PhD student |  | **√** | GAI self-evaluation | Current date confirmation | **√** | **√** | Task standardization |
| P 7 | Male | 26 | Salesperson | **√** | **√** | Environmental movie recommendations | Analysis of Japan’s economic situation | **√** | **√** | Frequency of errors |
| P 8 | Female | 23 | Master student | **√** | **√** | Playlist recommendations | Strategies to enhance competitiveness | **√** | **√** | GAI capability |
| P 9 | Female | 24 | Master student | **√** | **√** | Weight loss strategies | Methods to improve IELTS scores | **√** | **√** | Technological substitutability |
| P 10 | Female | 26 | Civil servant | **√** | **√** | Impact of hotpot on health checkup | Impact of hotpot on health checkup | **√** | **√** | Task urgency; frequency of errors |
| P 11 | Male | 23 | Master student |  | **√** | Introduction to black holes | Introduction to a certain enterprise |  |  | Perceived Recovery Expectations |
| P 12 | Female | 26 | Auditor |  | **√** | The writing of a tax audit report incorporating corporate risk | The writing of a tax audit report |  | **√** | Task urgency |
| P 13 | Male | 27 | Engineer | **√** | **√** | Specific strategies for learning large language models | Approaches to learning large language models |  | **√** | Task standardization |
| P 14 | Female | 26 | Civil servant | **√** | **√** | The writing of a rural revitalization research report | Current weather report | **√** | **√** | Task urgency |
| P 15 | Female | 26 | Bank clerk | **√** | **√** | Strategies to become self-disciplined | Strategies for managing complex workplace relationships | **√** | **√** |  |

**Appendix B. Interview Protocol.**

| Categories | Details |
| --- | --- |
| Basic Information | Age, gender, profession, adoption of ChatGPT |
| Expectation | What are your expectations for interacting with ChatGPT? |
|  |  |
| Evaluation (joint effect) | After experiencing successful functional anthropomorphism, which style of interaction would you prefer to continue: high-interactional anthropomorphism or low-interactional anthropomorphism? |
|  | After experiencing failed functional anthropomorphism, which style of interaction would you prefer to continue: high-interactional anthropomorphism or low-interactional anthropomorphism? |
| Mechanism | After ChatGPT’s functional failure, why would you choose to continue using (or not continue using) this high-interactional anthropomorphism GAI agent? |
| Boundary condition | When ChatGPT’s functional anthropomorphism fails, what key factors influence your attitude toward the effect of interactional anthropomorphism, and how do these factors influence your perspective? |

**Appendix C. The measurement of control variables.**

| Construct | Item | References |
| --- | --- | --- |
| Perceived cuteness | - This GAI lawyer is cute. | Lv et al., 2021 |
| Technological familiarity | - The technologies used in AI are familiar to me. | McDonough & Barczak, 1992 |
| Affinity for technology interaction | - I like to try new technologies. - I pay attention to the latest technological achievements. - I am curious about the development of new technologies. - I am willing to try new technological products. | Franke, Attig, & Wessel, 2019 |
| Trust of AI technology | - I believe that AI technology has sufficient capability to complete tasks. - I believe that AI technology has sufficient experience to complete tasks. - I believe that AI technology has sufficient resources to complete tasks. - I believe that AI technology can effectively complete tasks according to user needs. | Flavián, Guinalíu, & Gurrea, 2006 |

Notes: These items are measured using a 7-point Likert scale (1 = strongly disagree; 7 = strongly agree).

**Appendix D. Results of the joint effect in Experiment 1.**

| Variable | df | Mean square | F | Sig. |
| --- | --- | --- | --- | --- |
| Age | 1 | 0.078 | 0.043 | 0.836 |
| Gender | 1 | 0.019 | 0.011 | 0.918 |
| Affinity | 1 | 1.106 | 0.604 | 0.438 |
| Trust | 1 | 56.489 | 30.839 | 0.000 |
| Cuteness | 1 | 0.332 | 0.181 | 0.671 |
| Technological familiarity | 1 | 2.072 | 1.131 | 0.288 |
| Interactional anthropomorphism | 1 | 202.614 | 110.612 | 0.000 |
| Functional anthropomorphism | 1 | 18.354 | 10.020 | 0.002 |
| Interactional anthropomorphism * Functional anthropomorphism | 1 | 7.525 | 4.108 | 0.044 |

Notes: Dependent variable = Continuance intention.

**Appendix E. Results of the joint effect in Experiment 2.**

| Variable | df | Mean square | F | Sig. |
| --- | --- | --- | --- | --- |
| Age | 1 | 0.899 | 0.505 | 0.478 |
| Gender | 1 | 1.124 | 0.631 | 0.428 |
| Affinity | 1 | 7.166 | 4.021 | 0.046 |
| Trust | 1 | 42.738 | 23.984 | 0.000 |
| Cuteness | 1 | 0.059 | 0.033 | 0.856 |
| Technological familiarity | 1 | 0.122 | 0.068 | 0.794 |
| Interactional anthropomorphism | 1 | 204.976 | 115.030 | 0.000 |
| Functional anthropomorphism | 1 | 32.335 | 18.146 | 0.000 |
| Interactional anthropomorphism * Functional anthropomorphism | 1 | 8.644 | 4.851 | 0.028 |

Notes: Dependent variable = Continuance intention.

**Appendix F. The result of Experiment 4. Significance values.**

Notes: Significance values: ***p < 0.001, n.s.=not significant
